# Utilization of information from gene networks towards a better understanding of functional similarities between complex traits: a dairy cattle model

**DOI:** 10.1007/s13353-015-0306-5

**Published:** 2015-08-01

**Authors:** Magdalena Frąszczak, Tomasz Suchocki, Joanna Szyda

**Affiliations:** Biostatistics Group, Department of Genetics, Wroclaw University of Environmental and Life Sciences, Kożuchowska 7, 51-631 Wrocław, Poland

**Keywords:** Cattle, Distance metric, Gene networks, Genetic correlation, Genomic similarity, GO, Mixed model, SNP

## Abstract

Our study focused on quantifying functional similarities between complex traits recorded in dairy cattle: milk yield, fat yield, protein yield, somatic cell score and stature. Similarities were calculated based on gene sets forming gene networks and on gene ontology term sets underlying genes estimated as significant for the analysed traits. Gene networks were obtained by the Bisogenet and Gene Set Linkage Analysis (GSLA) software. The highest similarity was observed between milk yield and fat yield. A very low degree of similarity was attributed to protein yield and stature when using gene sets as a similarity criterion, as well as to protein yield and fat yield when using sets of gene ontology terms. Pearson correlation coefficients between gene effect estimates, representing additive polygenic similarities, were highest for protein yield and milk yield, and the lowest in case of protein yield and somatic cell score. Using the 50 K Illumina SNP chip from the national genomic selection data set only the most significant gene-trait associations can be retrieved, while enhancing it by the functional information contained in interaction data stored in public data bases and by metabolic pathways information facilitates a better characterization of the functional background of the traits and furthermore — trait comparison. The most interesting result of our study was that the functional similarity observed between protein yield and milk-/fat yields contradicted moderate genetic correlations estimated earlier for the same population based on a multivariate mixed model. The discrepancy indicates that an infinitesimal model assumed in that study reflects an averaged correlation due to polygenes, but fails to reveal the functional background underlying the traits, which is due to the cumulative composition of many genes involved in metabolic pathways, which appears to differ between protein-fat yield and protein-milk yield pairs.

**Electronic supplementary material:**

The online version of this article (doi:10.1007/s13353-015-0306-5) contains supplementary material, which is available to authorized users.

## Introduction

Recently in genetic analysis of complex traits the focus has been shifted from single genes identified via genome-wide association studies (GWAS) to genes identified via a functional analysis (Evangelou et al. 2014; Visscher et al. 2012). While genes selected by GWAS represent a selection of variants with (very) high effects on disease risk or on trait genetic variation, sets of genes selected by the functional approach are likely to also contain variants with moderate to small effects manifested through participation in important functional processes (Eleftherohorinou et al. 2009; Wang et al. 2010).

In our study we were interested in the incorporation of functional information from gene network analysis into the assessment of similarity between selected quantitative traits. This idea was first introduced by McGary et al. (2010), but later Woods et al. (2013) developed this concept to derive phenologs, i.e. phenotypes orthologous between species, e.g. by showing that mouse phenotypes — *clonic seizures* and *abnormal brain wave pattern* — are genomically similar to human epilepsy. Our study focussed on a within-species phenotype comparison by quantifying functional similarities between traits routinely recorded in dairy cattle. Recently, Pszczola et al. (2013) used the so-called predictor traits with widely available records in cattle populations, e.g. *fat–protein-corrected milk*, to enhance the accuracy of genomic prediction for other traits with less phenotypic information available such as, e.g. *dry matter intake*. Conceptually this can be considered as a within-species phenolog approach on an additive polygenic basis, i.e. with the underlying assumption of an infinitesimal mode of inheritance of phenotypes, with identification of neither particular genes nor the pathways. Our goal was to compare similarities between traits based on the functional information gathered through gene networks and thus assuming an underlying complex mode of inheritance.

## Materials and methods

### Material

Deregressed estimated breeding values predicted in the national routine evaluation of 2601 bulls from the Polish Holstein-Friesian dairy cattle breed were used in this analysis. Breeding values comprised production traits: milk-, fat- and protein yields (MKG, FKG, and PKG), somatic cell score (SCS), two type traits: stature (STA) and body size score (SIZ), as well as two fertility traits: non-return rate for heifers (NRH) and non-return rate for cows (NRC). All those traits undergo a complex mode of inheritance determined by major genes, as well as a large number of genes with moderate and low effects with heritabilities in the Polish population estimated at 0.33 for MKG, 0.29 for FKG and 0.29 for PKG, 0.32 for SCS, 0.54 for STA, 0.50 for SIZ, as well as 0.02 for NRH and NRC.

Genotypes comprise SNPs from the Illumina BovineSNP50 Genotyping BeadChip, which consists of 54,001 SNPs (version 1) and 54,609 SNPs (version 2). Genetic samples were provided within the frame of the MASinBULL project and comprised semen probes acquired via a routine semen collection procedure. Genotype preprocessing comprised elimination of SNPs with minor allele frequency below 0.01 and call rate under 90 % and resulted in 46,267 SNPs selected for the analysis.

### GWAS

Effects of the 46,267 SNPs were estimated using a SNP-BLUP model as described in Szyda et al. (2011). Statistically, this is a mixed model with random effects of SNPs described by a diagonal covariance matrix and bulls’ pseudophenotypes as dependent variables. Based on the estimated SNP effects, information of SNP genomic location and the pairwise linkage disequilibrium between SNPs, underlying gene effects were calculated and tested for significance using a normal approximation of the t-test, as described in detail by Szyda et al. (2012).

### Genomic and functional information

Genes showing effects significant with a maximum 20 % type I error rate were selected, separately for each trait, as scaffolds for the network construction. For better result validation two software packages were used to generate networks, i.e. the Bisogenet plugin (Martin et al. 2010) to the Cytoscape software (Shannon et al. 2003) and the stand alone Gene Set Linkage Analysis (GSLA) programme (Zhou et al. 2013). Both approaches construct networks of genes based on retrieving biological relations stored in multiple public data bases. For gene network generation Bisogenet utilizes data on protein-protein and protein-DNA interactions stored in publicly available data sets, as well as information from KEGG and signalling pathways. GSLA utilizes data on protein interactions predicted by the HIR V1 prediction model and 69,586 experimentally reported interactions. In both programmes the human data base was utilized, since interaction information for cattle available to date is very limited. The functional information was expressed either by sets of genes in generated networks or by the sets of gene ontology (GO) terms associated with the genes which were significant in GWAS.

### Genomic similarities

The sets of genes composing each network and the sets of GO terms associated with significant genes were summarized in a design matrix (Supporting information Table S1), which was then used to calculate similarity scores between traits. Two measures were used to quantify similarities between pairs of traits by comparing the sets of genes underlying networks for each trait and by comparing sets of GO terms related to genes, which effects were estimated as significant in GWAS analysis. The cosine similarity between traits *i* and *j* is given by: $\cos =\frac{N_{ij}}{N_i+{N}_j}$, where *N*_*ij*_ represents the number of times a feature (i.e. gene or GO term) was significant for both traits, *N*_*i*_*(N*_*j*_*)* is the number of times a feature was significant for trait *i(j)*. Spatially, the metric represents an angle between two vectors of features. The Jaccard similarity coefficient, defined as the quotient between the intersection and the union of the pairwise compared variables: $Jac=\frac{N_{ij}}{N_i+{N}_j+{N}_{ij}}$. In addition, Pearson correlation coefficients were calculated between SNP and gene effect estimates for each pair of traits.

## Results

### Genes

For size and non-return rate for cows and heifers no gene effect exceeded the 20 % significance threshold and thus the traits were not used for further analysis. For milk yield seven genes located on BTA14 were selected as significant, with effects ranging between 2.79 kg milk and 7.52 kg milk. For fat yield nine genes were selected, all located on BTA14, with effects between 0.11 kg fat and 0.39 kg fat. For protein yield six genes located on BTA03, BTA08, BTA17, BTA18, BTA19 and BTA29 were selected, with effects of 0.08 and 0.09 kg protein. Most genes (29), all with moderate standardized effects varying between 1.29 and 1.79, were selected for somatic cell score and were located on BTA01, BTA07, BTA09, BTA10, BTA12, BTA13, BTA17-20, BTA22-24 and BTA29. For stature two genes with standardized effects of 1.29 and 1.66 were selected on BTA5.

### Gene networks

The networks obtained by Bisogenet and GSLA for production traits consisted of 98 and 34 genes for MKG with 17.4 % of genes overlapping between both programmes, 97 and 64 genes for FKG (23.6 % overlap), as well as 44 and 87 genes for PKG (24.43 % overlap). The largest network consisting of 1255 and 1437 genes with a 32.4 % overlap between programmes was obtained for SCS and the smallest network was observed for STA with 26 and 59 genes (10.6 % overlap). The list of genes selected for the analysed traits, representing vectors used for the calculation of genomic similarities, is given in the Supporting information Table S1.

### Similarities between traits

**Fig. 1:**
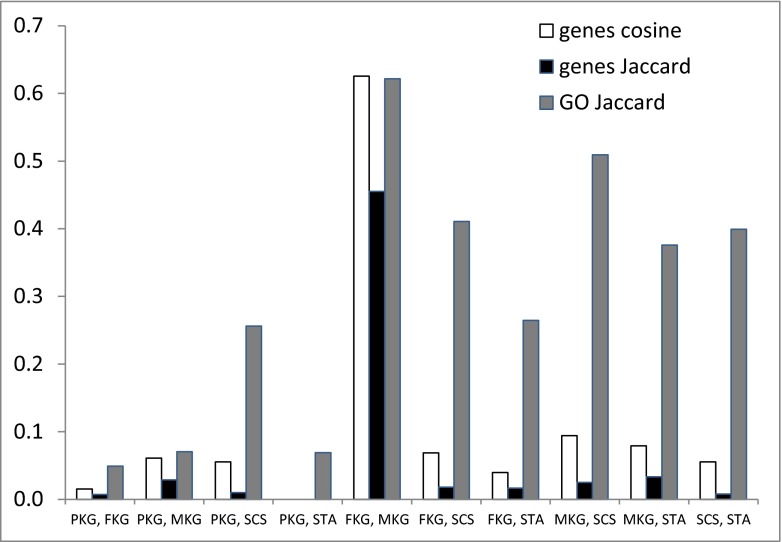
Similarity measures between traits calculated based on gene and GO term sets

**Fig. 2:**
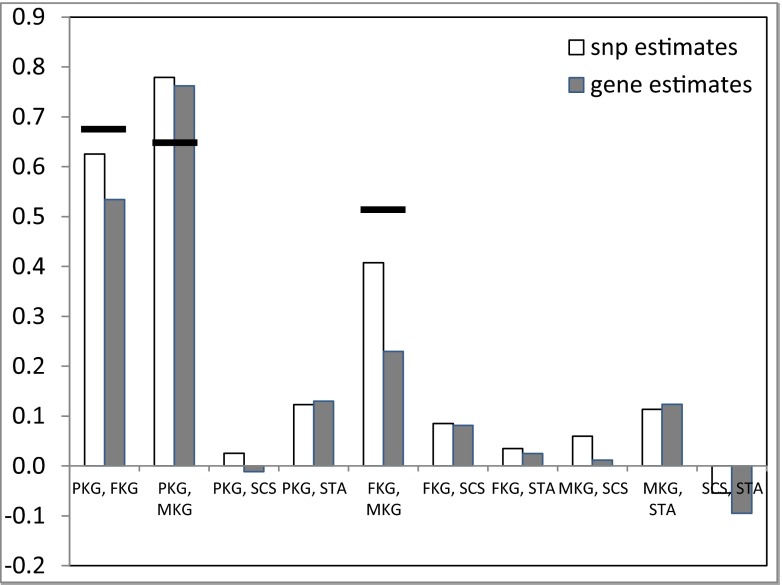
Pearson correlation coefficients between traits calculated based on gene and SNP effect estimates. Horisontal bars represent genetic correlations estimated by Jesiołkiewicz et al. (2011)

Similarities between traits based on gene and GO term sets underlying the gene networks, calculated using two different measures, i.e. the cosine and the Jaccard coefficients, were very consistent. While comparing sets of genes constituting a gene network for each trait the highest similarity of 0.455 was observed between MKG and FKG, while no similarity, expressed by metrics equal to 0, was observed between PKG and STA. Considering sets of GO terms characterizing the significant genes the highest similarity score of 0.622 was also calculated for MKG and FKG, while the lowest score of 0.049 was attributed to PKG and FKG (Fig. 1). Pearson correlation coefficients calculated between 4345 estimates of gene effects were highest for PKG and MKG (0.762) and lowest (−0.011) for PKG and SCS, whereas correlations between 46,267 SNP estimates ranged from 0.779 for PKG-MKG to 0.025 for PKG-STA (Fig. 2). When comparing the results it is noteworthy that for many trait-pair combinations polygenic based information expressed by Pearson correlation coefficients is not consistent with the functional similarity measures, particularly all of the trait pair comparisons involving PKG indicated high polygenic similarity, but low functional similarity.

## Discussion

The approach to derive functional information on complex phenotypes from GWAS, applied in this study, has already been postulated by Eleftherohorinou et al. (2009). Those authors stressed that using GWAS only the most significant gene-trait associations can be retrieved, which merely “represent the tip of the iceberg” of all potential genes involved in the determination of a quantitative phenotype, most of which have small individual effects. The impact of such genes on the determination of a complex trait is then manifested through their cumulative effect within functional pathways. This reasoning is exactly in line with our understanding of the genetic determination of complex traits and the corresponding methodology applied in our study attempts to extract most of the genomic background. A potential drawback of the experimental design of our study is connected with a relatively low coverage of the bovine genome by the 46,267 SNPs available for the analysis. The average intermarker distance was 51,728 bp, indicating some long gaps of the genomic sequence without SNP information. Therefore, out of over approximately 30,000 genes identified for dairy cattle we were able to pinpoint only 4345 with direct or closely located SNPs. Another aspect often neglected in association studies is that not all estimated significant associations may really represent physical linkage between a SNP and the genomic region. Some of the associations may arise through selection and the associated nonrandom mating in the population (Falconer and Mackay 1996). A technical limitation of the proposed approach results from the fact that it is based on GWAS results to select genes used as a scaffold for gene networks or GO terms. Since GWAS is only able to pinpoint genes of moderate to high effects on a quantitative trait variation, no scaffold can be created for traits with a pure polygenic (i.e. without major genes) mode of inheritance, in our case SIZ, NRH and NRC.

On the other hand, due to a very small effective population size in dairy cattle, linkage disequilibrium is very strong within 1000 bp of physical distance (Qanbari et al. 2010), assumed as a threshold distance between a SNP and a gene in our study, the 4345 gene effects are expected to be accurate even if most of the polymorphisms are not located within a gene and thus do not represent causal mutations. Moreover, a large sample size and a very low level of residual noise thanks to the bull pseudophenotypes used being a function of thousands of records further contribute to the accuracy of the results.

The apparently surprising result of our study is that the functional similarity observed between protein yield and milk/fat yield contradicts moderate genetic correlations estimated earlier for the same population based on a multivariate mixed model (Jesiołkiewicz et al. 2011). The discrepancy indicates that an infinitesimal model assumed in that study reflects an averaged correlation due to polygenes, but fails to reveal the functional background underlying the traits. As discussed by Shipley (2000) genes that are statistically the most significant are not necessarily direct, physiological causes of a complex phenotype. Consequently it appears that metabolic pathways underlying PKG and FKG/MKG appear to be different to a large extent. Such an outcome could have been expected when considering experimental results — e.g. Bauman and Griinari (2010) reported that the diet-induced low-fat milk syndrome in dairy cows does not affect protein yield, while food supplementation with biotin increased milk yield, but showed only a limited effect on milk composition (Girard and Matte 1988). On the other hand, similarities between PKG and FKG/MKG were reported in studies where an averaged genetic effect was considered, e.g. in a selection experiment reported by Kay et al. (2005) where selection on increased milk yield also resulted in an increased protein yield, or in studies focused on major genes with pleiotropic effects, e.g. DGAT1 reported to jointly influence MKG, PKG and FKG (Grisart et al. 2002). Polygenic based correlations between milk production traits (MKG, FKG, PKG) and SCS reported in the literature are very low, practically equal to zero (Miglior et al. 2007), which is in agreement with results estimated based on gene set similarity.

The two similarity measures provided very concordant results. However, the Jaccard metric based on gene sets was consistently lower than values based on GO term set based similarity. It should be noted here that GO terms were related to genes, which were significant in GWAS and thus represent only the most significant associations, whereas gene sets include information on interactions and thus provide a broader insight into the functional background of traits.

The major idea behind our study was to show that one does not need to rely only on “raw” information from gene effects estimated in GWAS or polygenic effects estimated in conventional mixed models, since they do not take into account other sources of biological information other than phenotype-genotype correlations. The mixture of the two sources of information, i.e. results of GWAS and functional information contained in public interaction data bases and metabolic pathways better characterizes the functional background of quantitative traits and furthermore facilitates their comparison.

## Electronic supplementary material

Supporting information Table S1 Matrix of genes constituting networks for the analysed traits used to calculate trait functional similarity measures. (DOCX 95 kb)
