# Supplementary material for: Utilization of information from gene networks towards a better understanding of functional similarities between complex traits: a dairy cattle model
Source: J Appl Genet. 2015 Aug 1;57:129–33. doi: 10.1007/s13353-015-0306-5 (PMC4731432; doi:10.1007/s13353-015-0306-5)
Supplement: Supplementary file 1 — Matrix of genes constituting networks for the analysed traits used to calculate trait functional similarity measures. (DOCX 95 kb) [file 13353_2015_306_MOESM1_ESM.docx]

**Table S1**

| **Gene**  **name** | **FKG** | **MKG** | **PKG** | **SCS** | **STA** |
| --- | --- | --- | --- | --- | --- |
| EGFR | 1 | 1 | 0 | 1 | 0 |
| NR3C1 | 1 | 1 | 0 | 1 | 0 |
| XPO1 | 1 | 0 | 0 | 0 | 0 |
| TNRC6C | 1 | 0 | 0 | 0 | 0 |
| USP20 | 1 | 1 | 0 | 1 | 0 |
| TOP2A | 1 | 0 | 0 | 0 | 0 |
| EZH2 | 1 | 0 | 0 | 0 | 0 |
| UBC | 1 | 1 | 1 | 1 | 0 |
| TNRC6A | 1 | 0 | 0 | 0 | 0 |
| CNKSR1 | 1 | 1 | 0 | 0 | 0 |
| TSC22D1 | 1 | 1 | 0 | 0 | 0 |
| DGAT1 | 1 | 1 | 0 | 0 | 0 |
| GABRA4 | 1 | 1 | 0 | 0 | 0 |
| TNF | 1 | 0 | 0 | 0 | 0 |
| CSNK2A2 | 1 | 1 | 0 | 0 | 0 |
| MAP2 | 1 | 1 | 0 | 1 | 0 |
| KCTD13 | 1 | 1 | 0 | 0 | 0 |
| ITGA6 | 1 | 1 | 0 | 1 | 0 |
| SPTA1 | 1 | 1 | 0 | 0 | 0 |
| ACTC1 | 1 | 1 | 0 | 0 | 0 |
| ABCB11 | 1 | 1 | 0 | 0 | 0 |
| CD1A | 1 | 1 | 0 | 0 | 0 |
| DHX9 | 1 | 0 | 0 | 0 | 0 |
| TBKBP1 | 1 | 0 | 0 | 0 | 0 |
| EIF2C4 | 1 | 0 | 0 | 0 | 0 |
| LRRK2 | 1 | 1 | 0 | 0 | 0 |
| EBS1 | 1 | 1 | 0 | 0 | 0 |
| EIF2C3 | 1 | 0 | 0 | 0 | 0 |
| PRKRA | 1 | 0 | 0 | 0 | 0 |
| PKIB | 1 | 1 | 0 | 0 | 0 |
| MAPK15 | 1 | 1 | 0 | 0 | 0 |
| TP53 | 1 | 0 | 0 | 1 | 0 |
| GIGYF2 | 1 | 0 | 0 | 0 | 0 |
| SUMO1 | 1 | 1 | 0 | 1 | 0 |
| NUDT3 | 1 | 1 | 0 | 0 | 0 |
| KRT15 | 1 | 1 | 0 | 1 | 0 |
| PLEKHA5 | 1 | 1 | 0 | 1 | 0 |
| GML | 1 | 0 | 0 | 0 | 0 |
| SRC | 1 | 1 | 0 | 1 | 1 |
| COL17A1 | 1 | 1 | 0 | 0 | 0 |
| GRB2 | 1 | 1 | 0 | 1 | 0 |
| ITGB4 | 1 | 1 | 0 | 1 | 0 |
| C14H8ORF33 | 1 | 1 | 0 | 0 | 0 |
| TNRC6B | 1 | 0 | 0 | 0 | 0 |
| CSK | 1 | 1 | 0 | 1 | 0 |
| MAF1 | 1 | 1 | 0 | 0 | 0 |
| CDK1 | 1 | 1 | 0 | 0 | 0 |
| ARMCX3 | 1 | 1 | 0 | 0 | 0 |
| IPO8 | 1 | 0 | 0 | 1 | 0 |
| GNB2L1 | 1 | 1 | 0 | 0 | 1 |
| RHOA | 1 | 1 | 0 | 0 | 0 |
| GEMIN4 | 1 | 0 | 0 | 0 | 0 |
| HBXIP | 1 | 0 | 0 | 0 | 0 |
| MSN | 1 | 1 | 0 | 0 | 0 |
| DCP1A | 1 | 0 | 0 | 0 | 0 |
| CLIC3 | 1 | 1 | 0 | 0 | 0 |
| CSNK2A1 | 1 | 1 | 0 | 1 | 0 |
| HTT | 1 | 0 | 0 | 1 | 0 |
| RBM4 | 1 | 0 | 0 | 0 | 0 |
| VIM | 1 | 1 | 0 | 1 | 0 |
| SMAD9 | 1 | 1 | 0 | 0 | 0 |
| MTOR | 1 | 1 | 0 | 0 | 0 |
| DDX6 | 1 | 0 | 0 | 0 | 0 |
| LMNB1 | 1 | 1 | 0 | 0 | 0 |
| DDX20 | 1 | 0 | 0 | 0 | 0 |
| TJP1 | 1 | 1 | 0 | 0 | 0 |
| CCND3 | 1 | 1 | 0 | 0 | 0 |
| CSNK2A1P | 1 | 0 | 0 | 0 | 0 |
| TGFB1I1 | 1 | 1 | 0 | 0 | 0 |
| RHPN1 | 1 | 1 | 0 | 0 | 0 |
| AGO2 | 1 | 0 | 0 | 0 | 0 |
| MTA2 | 1 | 0 | 0 | 0 | 0 |
| EIF4EBP1 | 1 | 0 | 0 | 0 | 0 |
| TP63 | 1 | 0 | 0 | 1 | 0 |
| ACTG1 | 1 | 1 | 0 | 0 | 0 |
| DICER1 | 1 | 0 | 0 | 0 | 0 |
| PFKP | 1 | 0 | 0 | 0 | 0 |
| TARBP2 | 1 | 0 | 0 | 0 | 0 |
| TBK1 | 1 | 0 | 0 | 0 | 0 |
| SRRM2 | 1 | 1 | 0 | 1 | 0 |
| MBP | 1 | 1 | 0 | 1 | 0 |
| EFEMP2 | 1 | 1 | 0 | 1 | 0 |
| SUMO2 | 1 | 1 | 0 | 1 | 0 |
| PCNA | 1 | 1 | 0 | 0 | 0 |
| EIF2C1 | 1 | 0 | 0 | 0 | 0 |
| DCP2 | 1 | 0 | 0 | 0 | 0 |
| ARFIP2 | 1 | 1 | 0 | 0 | 0 |
| DHX58 | 1 | 0 | 0 | 0 | 0 |
| TP73 | 1 | 0 | 0 | 0 | 0 |
| TRIM37 | 1 | 1 | 0 | 0 | 0 |
| CALCOCO2 | 1 | 1 | 0 | 0 | 0 |
| SPTAN1 | 1 | 1 | 0 | 0 | 0 |
| PAN2 | 1 | 1 | 0 | 0 | 0 |
| CASP8 | 1 | 1 | 0 | 0 | 0 |
| ETS1 | 1 | 1 | 0 | 0 | 0 |
| ROPN1 | 1 | 1 | 0 | 0 | 0 |
| LY6D | 1 | 1 | 0 | 1 | 0 |
| GIT1 | 0 | 0 | 0 | 1 | 0 |
| UPF1 | 0 | 0 | 0 | 1 | 0 |
| HSP90AA1 | 0 | 1 | 0 | 1 | 1 |
| RUVBL1 | 0 | 0 | 0 | 1 | 0 |
| CRK | 0 | 0 | 0 | 1 | 0 |
| LAMTOR5 | 0 | 1 | 0 | 0 | 0 |
| POLR1C | 0 | 0 | 0 | 0 | 0 |
| YWHAZ | 0 | 1 | 0 | 1 | 0 |
| VCP | 0 | 1 | 0 | 0 | 0 |
| VCAM1 | 0 | 1 | 0 | 1 | 0 |
| UQCRC1 | 0 | 1 | 0 | 0 | 0 |
| TSGA10 | 0 | 1 | 0 | 0 | 0 |
| TRA2A | 0 | 1 | 0 | 1 | 0 |
| SP1 | 0 | 1 | 0 | 1 | 0 |
| SNAPIN | 0 | 1 | 0 | 0 | 0 |
| SMC4 | 0 | 1 | 0 | 0 | 0 |
| SMC2 | 0 | 1 | 0 | 0 | 0 |
| SLTM | 0 | 1 | 0 | 0 | 0 |
| SIRT7 | 0 | 1 | 1 | 1 | 0 |
| RABEP1 | 0 | 1 | 0 | 0 | 0 |
| PPP1R3B | 0 | 1 | 0 | 0 | 0 |
| NOS2 | 0 | 1 | 0 | 0 | 0 |
| NOMO1 | 0 | 1 | 0 | 0 | 0 |
| NCAPH | 0 | 1 | 0 | 0 | 0 |
| NCAPG | 0 | 1 | 0 | 0 | 0 |
| MTDH | 0 | 1 | 0 | 0 | 0 |
| KIAA1967 | 0 | 1 | 0 | 0 | 0 |
| KIAA0101 | 0 | 1 | 0 | 1 | 0 |
| JUP | 0 | 1 | 0 | 0 | 0 |
| ITGA4 | 0 | 1 | 0 | 1 | 0 |
| ISG15 | 0 | 1 | 0 | 0 | 0 |
| HSP90AB1 | 0 | 1 | 0 | 1 | 1 |
| HNRNPL | 0 | 1 | 0 | 0 | 0 |
| GNAI2 | 0 | 1 | 0 | 0 | 0 |
| GBF1 | 0 | 1 | 0 | 0 | 0 |
| FN1 | 0 | 1 | 0 | 1 | 0 |
| FMNL1 | 0 | 1 | 0 | 0 | 0 |
| ESR1 | 0 | 1 | 0 | 1 | 0 |
| CUL3 | 0 | 1 | 1 | 1 | 0 |
| CSNK2A3 | 0 | 1 | 0 | 1 | 0 |
| CDK9 | 0 | 1 | 0 | 0 | 0 |
| CDK2 | 0 | 1 | 1 | 0 | 0 |
| AMPH | 0 | 0 | 1 | 0 | 0 |
| AP1B1 | 0 | 0 | 1 | 0 | 0 |
| AP1G1 | 0 | 0 | 1 | 0 | 0 |
| AP1M1 | 0 | 0 | 1 | 1 | 0 |
| AP1M2 | 0 | 0 | 1 | 0 | 0 |
| AP2M1 | 0 | 0 | 1 | 1 | 0 |
| ARF1 | 0 | 0 | 1 | 0 | 0 |
| ARF5 | 0 | 0 | 1 | 0 | 0 |
| ARF6 | 0 | 0 | 1 | 1 | 0 |
| ARR3 | 0 | 0 | 1 | 0 | 0 |
| ARRB2 | 0 | 0 | 1 | 0 | 0 |
| ATF7IP | 0 | 0 | 1 | 0 | 0 |
| ATM | 0 | 0 | 1 | 0 | 0 |
| ATR | 0 | 0 | 1 | 0 | 0 |
| BUB1 | 0 | 0 | 1 | 0 | 0 |
| BUB1B | 0 | 0 | 1 | 0 | 0 |
| CABIN1 | 0 | 0 | 1 | 0 | 0 |
| CHD3 | 0 | 0 | 1 | 0 | 0 |
| CLINT1 | 0 | 0 | 1 | 0 | 0 |
| CLTC | 0 | 0 | 1 | 1 | 0 |
| COPS5 | 0 | 0 | 1 | 0 | 0 |
| CREBBP | 0 | 0 | 1 | 0 | 0 |
| DDX34 | 0 | 0 | 1 | 0 | 0 |
| EPN1 | 0 | 0 | 1 | 0 | 0 |
| EPS15 | 0 | 0 | 1 | 1 | 0 |
| FBP2 | 0 | 0 | 1 | 0 | 0 |
| GSK3B | 0 | 0 | 1 | 1 | 0 |
| HEPHL1 | 0 | 0 | 1 | 0 | 0 |
| KIF13A | 0 | 0 | 1 | 1 | 0 |
| LHX7 | 0 | 0 | 1 | 0 | 0 |
| NTRK2 | 0 | 0 | 1 | 0 | 0 |
| PIP5K1C | 0 | 0 | 1 | 0 | 0 |
| POLA2 | 0 | 0 | 1 | 1 | 0 |
| PRKDC | 0 | 0 | 1 | 1 | 0 |
| RPS6KA6 | 0 | 0 | 1 | 0 | 0 |
| SHBG | 0 | 0 | 1 | 1 | 0 |
| SMAD2 | 0 | 0 | 1 | 0 | 0 |
| SNAP91 | 0 | 0 | 1 | 0 | 0 |
| SNCA | 0 | 0 | 1 | 0 | 0 |
| TANC2 | 0 | 0 | 1 | 0 | 0 |
| AP2S1 | 0 | 0 | 0 | 1 | 0 |
| AP2B1 | 0 | 0 | 0 | 1 | 0 |
| AP2A2 | 0 | 0 | 0 | 1 | 0 |
| HGS | 0 | 0 | 0 | 1 | 0 |
| COPB1 | 0 | 0 | 0 | 1 | 0 |
| POLR1B | 0 | 0 | 0 | 1 | 0 |
| GGA2 | 0 | 0 | 0 | 1 | 0 |
| SYNJ1 | 0 | 0 | 0 | 1 | 0 |
| MLST8 | 0 | 0 | 0 | 1 | 0 |
| PSMB3 | 0 | 0 | 0 | 1 | 0 |
| WBSCR16 | 0 | 0 | 0 | 1 | 0 |
| CALM2 | 0 | 0 | 0 | 1 | 0 |
| VLDLR | 0 | 0 | 0 | 1 | 0 |
| SERPINH1 | 0 | 0 | 0 | 1 | 0 |
| SETD2 | 0 | 0 | 0 | 1 | 0 |
| CFH | 0 | 0 | 0 | 1 | 0 |
| ARID5A | 0 | 0 | 0 | 1 | 0 |
| ARAF | 0 | 0 | 0 | 1 | 0 |
| PIAS1 | 0 | 0 | 0 | 1 | 0 |
| CD2AP | 0 | 0 | 0 | 1 | 0 |
| PAK4 | 0 | 0 | 0 | 1 | 0 |
| CCDC136 | 0 | 0 | 0 | 1 | 0 |
| RASGRF2 | 0 | 0 | 0 | 1 | 0 |
| SHB | 0 | 0 | 0 | 1 | 0 |
| KIAA1549L | 0 | 0 | 0 | 1 | 0 |
| CYFIP2 | 0 | 0 | 0 | 1 | 0 |
| EPHA2 | 0 | 0 | 0 | 1 | 0 |
| EHMT2 | 0 | 0 | 0 | 1 | 0 |
| KCTD15 | 0 | 0 | 0 | 1 | 0 |
| EIF3D | 0 | 0 | 0 | 1 | 0 |
| KRT34 | 0 | 0 | 0 | 1 | 0 |
| RYR1 | 0 | 0 | 0 | 1 | 0 |
| SPATA2 | 0 | 0 | 0 | 1 | 0 |
| PRKAB1 | 0 | 0 | 0 | 1 | 0 |
| PSMC3 | 0 | 0 | 0 | 1 | 0 |
| GRB7 | 0 | 0 | 0 | 1 | 0 |
| LPAR2 | 0 | 0 | 0 | 1 | 0 |
| CYTH2 | 0 | 0 | 0 | 1 | 0 |
| SNX18 | 0 | 0 | 0 | 1 | 0 |
| TSC1 | 0 | 0 | 0 | 1 | 0 |
| TIAF1 | 0 | 0 | 0 | 1 | 0 |
| SLX1B | 0 | 0 | 0 | 1 | 0 |
| PHACTR4 | 0 | 0 | 0 | 1 | 0 |
| ASB16 | 0 | 0 | 0 | 1 | 0 |
| TSG101 | 0 | 0 | 0 | 1 | 0 |
| BIN1 | 0 | 0 | 0 | 1 | 0 |
| ND4 | 0 | 0 | 0 | 1 | 0 |
| CHMP2A | 0 | 0 | 0 | 1 | 0 |
| MDM2 | 0 | 0 | 0 | 1 | 0 |
| REPS2 | 0 | 0 | 0 | 1 | 0 |
| SNRPA | 0 | 0 | 0 | 1 | 0 |
| PTPN11 | 0 | 0 | 0 | 1 | 0 |
| HCLS1 | 0 | 0 | 0 | 1 | 0 |
| KRT7 | 0 | 0 | 0 | 1 | 0 |
| FRS3 | 0 | 0 | 0 | 1 | 0 |
| WASF2 | 0 | 0 | 0 | 1 | 0 |
| TUBB4B | 0 | 0 | 0 | 1 | 0 |
| SHC1 | 0 | 0 | 0 | 1 | 0 |
| SMARCD3 | 0 | 0 | 0 | 1 | 0 |
| NCK1 | 0 | 0 | 0 | 1 | 0 |
| ST6GALNAC6 | 0 | 0 | 0 | 1 | 0 |
| ZNF32 | 0 | 0 | 0 | 1 | 0 |
| C19H17ORF62 | 0 | 0 | 0 | 1 | 0 |
| MED14 | 0 | 0 | 0 | 1 | 0 |
| TNPO1 | 0 | 0 | 0 | 1 | 0 |
| MYL12B | 0 | 0 | 0 | 1 | 0 |
| RAB5B | 0 | 0 | 0 | 1 | 0 |
| ILF3 | 0 | 0 | 0 | 1 | 0 |
| RBM14 | 0 | 0 | 0 | 1 | 0 |
| FLT4 | 0 | 0 | 0 | 1 | 0 |
| IL2RG | 0 | 0 | 0 | 1 | 0 |
| RAPSN | 0 | 0 | 0 | 1 | 0 |
| MYH11 | 0 | 0 | 0 | 1 | 0 |
| TRMT112 | 0 | 0 | 0 | 1 | 0 |
| TOM1L1 | 0 | 0 | 0 | 1 | 0 |
| SOS2 | 0 | 0 | 0 | 1 | 0 |
| GSPT1 | 0 | 0 | 0 | 1 | 0 |
| SKIV2L2 | 0 | 0 | 0 | 1 | 0 |
| LOC100653049 | 0 | 0 | 0 | 1 | 0 |
| UBA1 | 0 | 0 | 0 | 1 | 0 |
| CRYBB3 | 0 | 0 | 0 | 1 | 0 |
| CDC42 | 0 | 0 | 0 | 1 | 0 |
| LAT2 | 0 | 0 | 0 | 1 | 0 |
| ZHX1 | 0 | 0 | 0 | 1 | 0 |
| SLC12A2 | 0 | 0 | 0 | 1 | 0 |
| SOCS7 | 0 | 0 | 0 | 1 | 0 |
| SEC13 | 0 | 0 | 0 | 1 | 0 |
| CCT5 | 0 | 0 | 0 | 1 | 0 |
| NUDT21 | 0 | 0 | 0 | 1 | 0 |
| ALOX5 | 0 | 0 | 0 | 1 | 0 |
| IL5RA | 0 | 0 | 0 | 1 | 0 |
| CDIP1 | 0 | 0 | 0 | 1 | 0 |
| SHANK2 | 0 | 0 | 0 | 1 | 0 |
| APCS | 0 | 0 | 0 | 1 | 0 |
| LAMP1 | 0 | 0 | 0 | 1 | 0 |
| UBA52 | 0 | 0 | 0 | 1 | 0 |
| SH2B1 | 0 | 0 | 0 | 1 | 0 |
| ATP13A2 | 0 | 0 | 0 | 1 | 0 |
| PEX13 | 0 | 0 | 0 | 1 | 0 |
| MERTK | 0 | 0 | 0 | 1 | 0 |
| SMARCD1 | 0 | 0 | 0 | 1 | 0 |
| RP1L1 | 0 | 0 | 0 | 1 | 0 |
| ZNF259 | 0 | 0 | 0 | 1 | 0 |
| BAD | 0 | 0 | 0 | 1 | 0 |
| TNK2 | 0 | 0 | 0 | 1 | 1 |
| DZIP3 | 0 | 0 | 0 | 1 | 0 |
| MYLIP | 0 | 0 | 0 | 1 | 0 |
| KIF26A | 0 | 0 | 0 | 1 | 0 |
| C1orf27 | 0 | 0 | 0 | 1 | 0 |
| ZNF804A | 0 | 0 | 0 | 1 | 0 |
| CAND1 | 0 | 0 | 0 | 1 | 0 |
| CLCN2 | 0 | 0 | 0 | 1 | 0 |
| RET | 0 | 0 | 0 | 1 | 0 |
| R3HDM2 | 0 | 0 | 0 | 1 | 0 |
| SPTBN1 | 0 | 0 | 0 | 1 | 0 |
| ITK | 0 | 0 | 0 | 1 | 0 |
| B2M | 0 | 0 | 0 | 1 | 0 |
| CRKL | 0 | 0 | 0 | 1 | 0 |
| BABAM1 | 0 | 0 | 0 | 1 | 0 |
| HIST1H4B | 0 | 0 | 0 | 1 | 0 |
| ENTPD6 | 0 | 0 | 0 | 1 | 0 |
| CDV3 | 0 | 0 | 0 | 1 | 0 |
| FASLG | 0 | 0 | 0 | 1 | 0 |
| UBE2D4 | 0 | 0 | 0 | 1 | 0 |
| YWHAQ | 0 | 0 | 0 | 1 | 0 |
| PTPN6 | 0 | 0 | 0 | 1 | 0 |
| HDAC4 | 0 | 0 | 0 | 1 | 0 |
| CDC27 | 0 | 0 | 0 | 1 | 0 |
| SYNJ2 | 0 | 0 | 0 | 1 | 0 |
| HLA-B | 0 | 0 | 0 | 1 | 0 |
| ASNS | 0 | 0 | 0 | 1 | 0 |
| KRT85 | 0 | 0 | 0 | 1 | 0 |
| NTRK1 | 0 | 0 | 0 | 1 | 0 |
| SFPQ | 0 | 0 | 0 | 1 | 0 |
| MAPK9 | 0 | 0 | 0 | 1 | 0 |
| SKAP2 | 0 | 0 | 0 | 1 | 0 |
| POLR2A | 0 | 0 | 0 | 1 | 0 |
| RBM10 | 0 | 0 | 0 | 1 | 0 |
| TLE1 | 0 | 0 | 0 | 1 | 0 |
| GTF3C1 | 0 | 0 | 0 | 1 | 0 |
| SS18 | 0 | 0 | 0 | 1 | 0 |
| LRRC59 | 0 | 0 | 0 | 1 | 0 |
| TPX2 | 0 | 0 | 0 | 1 | 0 |
| TYMS | 0 | 0 | 0 | 1 | 0 |
| ATP1A3 | 0 | 0 | 0 | 1 | 0 |
| PTPN23 | 0 | 0 | 0 | 1 | 0 |
| NFYB | 0 | 0 | 0 | 1 | 0 |
| LRRK1 | 0 | 0 | 0 | 1 | 0 |
| MCM4 | 0 | 0 | 0 | 1 | 0 |
| HIST1H4A | 0 | 0 | 0 | 1 | 0 |
| PTK2 | 0 | 0 | 0 | 1 | 0 |
| KRT1 | 0 | 0 | 0 | 1 | 0 |
| VPS13A | 0 | 0 | 0 | 1 | 0 |
| NDUFA7 | 0 | 0 | 0 | 1 | 0 |
| RBM12B | 0 | 0 | 0 | 1 | 0 |
| AKT1 | 0 | 0 | 0 | 1 | 0 |
| INSR | 0 | 0 | 0 | 1 | 0 |
| HIST1H3J | 0 | 0 | 0 | 1 | 0 |
| EIF2B5 | 0 | 0 | 0 | 1 | 0 |
| GHR | 0 | 0 | 0 | 1 | 0 |
| ISG20L2 | 0 | 0 | 0 | 1 | 0 |
| IST1 | 0 | 0 | 0 | 1 | 0 |
| EFS | 0 | 0 | 0 | 1 | 0 |
| RBPJ | 0 | 0 | 0 | 1 | 0 |
| TRAIP | 0 | 0 | 0 | 1 | 0 |
| R3HDM1 | 0 | 0 | 0 | 1 | 0 |
| PATL1 | 0 | 0 | 0 | 1 | 0 |
| LEPR | 0 | 0 | 0 | 1 | 0 |
| AGER | 0 | 0 | 0 | 1 | 0 |
| SV2A | 0 | 0 | 0 | 1 | 0 |
| KRT17 | 0 | 0 | 0 | 1 | 0 |
| KRT19 | 0 | 0 | 0 | 1 | 0 |
| MTPN | 0 | 0 | 0 | 1 | 0 |
| ATXN2L | 0 | 0 | 0 | 1 | 0 |
| DNM3 | 0 | 0 | 0 | 1 | 0 |
| ADA | 0 | 0 | 0 | 1 | 0 |
| SNRNP200 | 0 | 0 | 0 | 1 | 0 |
| PIK3AP1 | 0 | 0 | 0 | 1 | 0 |
| PTPN4 | 0 | 0 | 0 | 1 | 0 |
| DIXDC1 | 0 | 0 | 0 | 1 | 0 |
| TSC2 | 0 | 0 | 0 | 1 | 0 |
| GJA9-MYCBP | 0 | 0 | 0 | 1 | 0 |
| TUB | 0 | 0 | 0 | 1 | 0 |
| CAST | 0 | 0 | 0 | 1 | 0 |
| EIF2S2 | 0 | 0 | 0 | 1 | 0 |
| EP300 | 0 | 0 | 0 | 1 | 0 |
| SRSF1 | 0 | 0 | 0 | 1 | 0 |
| NPHP3 | 0 | 0 | 0 | 1 | 0 |
| PSMA5 | 0 | 0 | 0 | 1 | 0 |
| SNX12 | 0 | 0 | 0 | 1 | 0 |
| STAC2 | 0 | 0 | 0 | 1 | 0 |
| MDC1 | 0 | 0 | 0 | 1 | 0 |
| LDB1 | 0 | 0 | 0 | 1 | 0 |
| ZC3H10 | 0 | 0 | 0 | 1 | 0 |
| WNK1 | 0 | 0 | 0 | 1 | 0 |
| EGF | 0 | 0 | 0 | 1 | 0 |
| LAT | 0 | 0 | 0 | 1 | 0 |
| ANK2 | 0 | 0 | 0 | 1 | 0 |
| NCSTN | 0 | 0 | 0 | 1 | 0 |
| PTPN1 | 0 | 0 | 0 | 1 | 0 |
| MLL | 0 | 0 | 0 | 1 | 0 |
| MCM7 | 0 | 0 | 0 | 1 | 0 |
| BRE | 0 | 0 | 0 | 1 | 0 |
| PDGFRA | 0 | 0 | 0 | 1 | 0 |
| DNM1 | 0 | 0 | 0 | 1 | 0 |
| PTGDS | 0 | 0 | 0 | 1 | 0 |
| HSD17B10 | 0 | 0 | 0 | 1 | 0 |
| ZKSCAN3 | 0 | 0 | 0 | 1 | 0 |
| DLG4 | 0 | 0 | 0 | 1 | 1 |
| CENPN | 0 | 0 | 0 | 1 | 0 |
| SRSF7 | 0 | 0 | 0 | 1 | 0 |
| TNFRSF14 | 0 | 0 | 0 | 1 | 0 |
| ZNF585B | 0 | 0 | 0 | 1 | 0 |
| BRPF3 | 0 | 0 | 0 | 1 | 0 |
| PRPF6 | 0 | 0 | 0 | 1 | 0 |
| GCFC2 | 0 | 0 | 0 | 1 | 0 |
| IGFLR1 | 0 | 0 | 0 | 1 | 0 |
| IMMT | 0 | 0 | 0 | 1 | 0 |
| HPN | 0 | 0 | 0 | 1 | 0 |
| CCT8 | 0 | 0 | 0 | 1 | 0 |
| AEBP1 | 0 | 0 | 0 | 1 | 0 |
| ASAP2 | 0 | 0 | 0 | 1 | 0 |
| PANX2 | 0 | 0 | 0 | 1 | 0 |
| MRPL53 | 0 | 0 | 0 | 1 | 0 |
| KHSRP | 0 | 0 | 0 | 1 | 0 |
| FABP1 | 0 | 0 | 0 | 1 | 0 |
| UBE2E4P | 0 | 0 | 0 | 1 | 0 |
| C9H6ORF170 | 0 | 0 | 0 | 1 | 0 |
| EIF4ENIF1 | 0 | 0 | 0 | 1 | 0 |
| RPS6KA1 | 0 | 0 | 0 | 1 | 0 |
| C3ORF21 | 0 | 0 | 0 | 1 | 0 |
| YWHAG | 0 | 0 | 0 | 1 | 0 |
| MAPK12 | 0 | 0 | 0 | 1 | 0 |
| ACTB | 0 | 0 | 0 | 1 | 0 |
| GAS7 | 0 | 0 | 0 | 1 | 0 |
| SNTA1 | 0 | 0 | 0 | 1 | 0 |
| MPG | 0 | 0 | 0 | 1 | 0 |
| GAB1 | 0 | 0 | 0 | 1 | 0 |
| FOS | 0 | 0 | 0 | 1 | 0 |
| ST5 | 0 | 0 | 0 | 1 | 0 |
| SAR1A | 0 | 0 | 0 | 1 | 0 |
| CLEC4F | 0 | 0 | 0 | 1 | 0 |
| WAS | 0 | 0 | 0 | 1 | 0 |
| RACGAP1 | 0 | 0 | 0 | 1 | 0 |
| GPATCH8 | 0 | 0 | 0 | 1 | 0 |
| SH2D4A | 0 | 0 | 0 | 1 | 0 |
| DMXL2 | 0 | 0 | 0 | 1 | 0 |
| HGF | 0 | 0 | 0 | 1 | 0 |
| VPS4B | 0 | 0 | 0 | 1 | 0 |
| TOX4 | 0 | 0 | 0 | 1 | 0 |
| PDS5A | 0 | 0 | 0 | 1 | 0 |
| SNRPD1 | 0 | 0 | 0 | 1 | 0 |
| SLC25A13 | 0 | 0 | 0 | 1 | 0 |
| BEND2 | 0 | 0 | 0 | 1 | 0 |
| HIST1H3G | 0 | 0 | 0 | 1 | 0 |
| TRIM38 | 0 | 0 | 0 | 1 | 0 |
| FH | 0 | 0 | 0 | 1 | 0 |
| IGFL1 | 0 | 0 | 0 | 1 | 0 |
| YWHAE | 0 | 0 | 0 | 1 | 0 |
| WDR1 | 0 | 0 | 0 | 1 | 0 |
| UHRF1BP1L | 0 | 0 | 0 | 1 | 0 |
| MLXIPL | 0 | 0 | 0 | 1 | 0 |
| ALK | 0 | 0 | 0 | 1 | 0 |
| ZSWIM8 | 0 | 0 | 0 | 1 | 0 |
| AKTIP | 0 | 0 | 0 | 1 | 0 |
| HSPA1B | 0 | 0 | 0 | 1 | 0 |
| MAPK14 | 0 | 0 | 0 | 1 | 0 |
| OAZ1 | 0 | 0 | 0 | 1 | 0 |
| UGP2 | 0 | 0 | 0 | 1 | 0 |
| TTLL12 | 0 | 0 | 0 | 1 | 0 |
| DNM2 | 0 | 0 | 0 | 1 | 0 |
| SPRYD4 | 0 | 0 | 0 | 1 | 0 |
| MST1R | 0 | 0 | 0 | 1 | 0 |
| GSTZ1 | 0 | 0 | 0 | 1 | 0 |
| SSBP1 | 0 | 0 | 0 | 1 | 0 |
| DYNC1H1 | 0 | 0 | 0 | 1 | 0 |
| STAMBP | 0 | 0 | 0 | 1 | 0 |
| HNRNPC | 0 | 0 | 0 | 1 | 0 |
| UBE2E1 | 0 | 0 | 0 | 1 | 0 |
| SH2D5 | 0 | 0 | 0 | 1 | 0 |
| TCEA1P2 | 0 | 0 | 0 | 1 | 0 |
| PDE4DIP | 0 | 0 | 0 | 1 | 0 |
| HDLBP | 0 | 0 | 0 | 1 | 0 |
| PIK3CB | 0 | 0 | 0 | 1 | 0 |
| KHDRBS2 | 0 | 0 | 0 | 1 | 0 |
| ASB15 | 0 | 0 | 0 | 1 | 0 |
| PAXIP1 | 0 | 0 | 0 | 1 | 0 |
| SLCO2A1 | 0 | 0 | 0 | 1 | 0 |
| KIF3A | 0 | 0 | 0 | 1 | 0 |
| MS4A2 | 0 | 0 | 0 | 1 | 0 |
| CALD1 | 0 | 0 | 0 | 1 | 0 |
| WWOX | 0 | 0 | 0 | 1 | 0 |
| PRRC2B | 0 | 0 | 0 | 1 | 0 |
| ESRRA | 0 | 0 | 0 | 1 | 0 |
| MAP4K5 | 0 | 0 | 0 | 1 | 0 |
| KLHL12 | 0 | 0 | 0 | 1 | 0 |
| ACTG2 | 0 | 0 | 0 | 1 | 0 |
| CRMP1 | 0 | 0 | 0 | 1 | 0 |
| HIST1H3H | 0 | 0 | 0 | 1 | 0 |
| PTPRE | 0 | 0 | 0 | 1 | 1 |
| SLC25A6 | 0 | 0 | 0 | 1 | 0 |
| VPS18 | 0 | 0 | 0 | 1 | 0 |
| HIST2H4A | 0 | 0 | 0 | 1 | 0 |
| FTSJ1 | 0 | 0 | 0 | 1 | 0 |
| PIK3R1 | 0 | 0 | 0 | 1 | 0 |
| ACACA | 0 | 0 | 0 | 1 | 0 |
| NLK | 0 | 0 | 0 | 1 | 0 |
| MTA1 | 0 | 0 | 0 | 1 | 0 |
| NCL | 0 | 0 | 0 | 1 | 0 |
| FAU | 0 | 0 | 0 | 1 | 0 |
| ARHGEF11 | 0 | 0 | 0 | 1 | 0 |
| CLASP2 | 0 | 0 | 0 | 1 | 0 |
| GABBR1 | 0 | 0 | 0 | 1 | 0 |
| SNX3 | 0 | 0 | 0 | 1 | 0 |
| TUBB | 0 | 0 | 0 | 1 | 0 |
| CYP4F2 | 0 | 0 | 0 | 1 | 0 |
| KPNA2 | 0 | 0 | 0 | 1 | 0 |
| IGBP1 | 0 | 0 | 0 | 1 | 0 |
| VHL | 0 | 0 | 0 | 1 | 0 |
| STARD10 | 0 | 0 | 0 | 1 | 0 |
| EIF2S1 | 0 | 0 | 0 | 1 | 0 |
| EXTL1 | 0 | 0 | 0 | 1 | 0 |
| PPP1CC | 0 | 0 | 0 | 1 | 0 |
| UNC119 | 0 | 0 | 0 | 1 | 0 |
| RIT1 | 0 | 0 | 0 | 1 | 0 |
| ACTL6A | 0 | 0 | 0 | 1 | 0 |
| TDP2 | 0 | 0 | 0 | 1 | 0 |
| CELSR3 | 0 | 0 | 0 | 1 | 0 |
| ERBB3 | 0 | 0 | 0 | 1 | 0 |
| RBP4 | 0 | 0 | 0 | 1 | 0 |
| USP7 | 0 | 0 | 0 | 1 | 0 |
| SMYD2 | 0 | 0 | 0 | 1 | 0 |
| ZYX | 0 | 0 | 0 | 1 | 0 |
| DTX1 | 0 | 0 | 0 | 1 | 0 |
| HNRNPK | 0 | 0 | 0 | 1 | 0 |
| NCKAP5 | 0 | 0 | 0 | 1 | 0 |
| DCC | 0 | 0 | 0 | 1 | 0 |
| PDE4D | 0 | 0 | 0 | 1 | 0 |
| ZNF326 | 0 | 0 | 0 | 1 | 0 |
| ATXN1 | 0 | 0 | 0 | 1 | 0 |
| LDLR | 0 | 0 | 0 | 1 | 0 |
| FAM110A | 0 | 0 | 0 | 1 | 0 |
| PRPF8 | 0 | 0 | 0 | 1 | 0 |
| MYC | 0 | 0 | 0 | 1 | 0 |
| CHMP1A | 0 | 0 | 0 | 1 | 0 |
| PSMD1 | 0 | 0 | 0 | 1 | 0 |
| HIST1H4D | 0 | 0 | 0 | 1 | 0 |
| STAM2 | 0 | 0 | 0 | 1 | 0 |
| NKX2-1 | 0 | 0 | 0 | 1 | 0 |
| HIST1H4K | 0 | 0 | 0 | 1 | 0 |
| IARS | 0 | 0 | 0 | 1 | 0 |
| PSMD14 | 0 | 0 | 0 | 1 | 0 |
| DOCK5 | 0 | 0 | 0 | 1 | 0 |
| PIK3C2B | 0 | 0 | 0 | 1 | 0 |
| TNFRSF10D | 0 | 0 | 0 | 1 | 0 |
| C1orf94 | 0 | 0 | 0 | 1 | 0 |
| BCL2A1 | 0 | 0 | 0 | 1 | 0 |
| KRT5 | 0 | 0 | 0 | 1 | 0 |
| TSKS | 0 | 0 | 0 | 1 | 0 |
| IL2RB | 0 | 0 | 0 | 1 | 0 |
| SHC3 | 0 | 0 | 0 | 1 | 0 |
| HPCAL1 | 0 | 0 | 0 | 1 | 0 |
| C20orf57 | 0 | 0 | 0 | 1 | 0 |
| VAV3 | 0 | 0 | 0 | 1 | 0 |
| OTUD5 | 0 | 0 | 0 | 1 | 0 |
| CDKL5 | 0 | 0 | 0 | 1 | 0 |
| EPHB1 | 0 | 0 | 0 | 1 | 0 |
| HUNK | 0 | 0 | 0 | 1 | 0 |
| APOH | 0 | 0 | 0 | 1 | 0 |
| FTL | 0 | 0 | 0 | 1 | 0 |
| PON2 | 0 | 0 | 0 | 1 | 0 |
| KAT5 | 0 | 0 | 0 | 1 | 0 |
| NPHP3-ACAD11 | 0 | 0 | 0 | 1 | 0 |
| GMEB2 | 0 | 0 | 0 | 1 | 0 |
| PIM2 | 0 | 0 | 0 | 1 | 0 |
| KRT81 | 0 | 0 | 0 | 1 | 0 |
| MAP1A | 0 | 0 | 0 | 1 | 0 |
| MAP3K7 | 0 | 0 | 0 | 1 | 0 |
| ADRB1 | 0 | 0 | 0 | 1 | 0 |
| ADAM12 | 0 | 0 | 0 | 1 | 0 |
| SUPT20H | 0 | 0 | 0 | 1 | 0 |
| TP53I11 | 0 | 0 | 0 | 1 | 0 |
| CNPY2 | 0 | 0 | 0 | 1 | 0 |
| KLF11 | 0 | 0 | 0 | 1 | 0 |
| KCNH7 | 0 | 0 | 0 | 1 | 0 |
| RBMX | 0 | 0 | 0 | 1 | 0 |
| RPL13 | 0 | 0 | 0 | 1 | 0 |
| AXL | 0 | 0 | 0 | 1 | 0 |
| TRIM39 | 0 | 0 | 0 | 1 | 0 |
| PTPN18 | 0 | 0 | 0 | 1 | 0 |
| SCARF2 | 0 | 0 | 0 | 1 | 0 |
| ZHX2 | 0 | 0 | 0 | 1 | 0 |
| ADAM10 | 0 | 0 | 0 | 1 | 0 |
| KIAA1377 | 0 | 0 | 0 | 1 | 0 |
| HEYL | 0 | 0 | 0 | 1 | 0 |
| ESD | 0 | 0 | 0 | 1 | 0 |
| FCGR2B | 0 | 0 | 0 | 1 | 0 |
| CDK6 | 0 | 0 | 0 | 1 | 0 |
| PTPN12 | 0 | 0 | 0 | 1 | 0 |
| CTAGE5 | 0 | 0 | 0 | 1 | 0 |
| CHMP1B | 0 | 0 | 0 | 1 | 0 |
| PHC2 | 0 | 0 | 0 | 1 | 0 |
| TRIM41 | 0 | 0 | 0 | 1 | 0 |
| VPS16 | 0 | 0 | 0 | 1 | 0 |
| GABARAPL1 | 0 | 0 | 0 | 1 | 0 |
| ILK | 0 | 0 | 0 | 1 | 0 |
| KCNAB2 | 0 | 0 | 0 | 1 | 0 |
| PRDM1 | 0 | 0 | 0 | 1 | 0 |
| TTC19 | 0 | 0 | 0 | 1 | 0 |
| KRT16 | 0 | 0 | 0 | 1 | 0 |
| SUGP1 | 0 | 0 | 0 | 1 | 0 |
| VASN | 0 | 0 | 0 | 1 | 0 |
| CD19 | 0 | 0 | 0 | 1 | 0 |
| HSPA9 | 0 | 0 | 0 | 1 | 0 |
| YY1AP1 | 0 | 0 | 0 | 1 | 0 |
| MAP1LC3C | 0 | 0 | 0 | 1 | 0 |
| POLD1 | 0 | 0 | 0 | 1 | 0 |
| HSPA5 | 0 | 0 | 0 | 1 | 0 |
| SHROOM2 | 0 | 0 | 0 | 1 | 0 |
| SF3A1 | 0 | 0 | 0 | 1 | 0 |
| CALM1 | 0 | 0 | 0 | 1 | 0 |
| CHRM4 | 0 | 0 | 0 | 1 | 0 |
| TAB1 | 0 | 0 | 0 | 1 | 0 |
| GAB2 | 0 | 0 | 0 | 1 | 0 |
| NEK8 | 0 | 0 | 0 | 1 | 0 |
| DHX15 | 0 | 0 | 0 | 1 | 0 |
| CD28 | 0 | 0 | 0 | 1 | 0 |
| UBXN1 | 0 | 0 | 0 | 1 | 0 |
| CD247 | 0 | 0 | 0 | 1 | 0 |
| OXSR1 | 0 | 0 | 0 | 1 | 0 |
| PTPN22 | 0 | 0 | 0 | 1 | 0 |
| CCDC6 | 0 | 0 | 0 | 1 | 0 |
| WASL | 0 | 0 | 0 | 1 | 0 |
| GCN1L1 | 0 | 0 | 0 | 1 | 0 |
| ZBTB32 | 0 | 0 | 0 | 1 | 0 |
| DCUN1D1 | 0 | 0 | 0 | 1 | 0 |
| GRB10 | 0 | 0 | 0 | 1 | 0 |
| GAPDH | 0 | 0 | 0 | 1 | 0 |
| PPP1CA | 0 | 0 | 0 | 1 | 0 |
| CETN2 | 0 | 0 | 0 | 1 | 0 |
| SVIP | 0 | 0 | 0 | 1 | 0 |
| CXCR7 | 0 | 0 | 0 | 1 | 0 |
| HIST1H4L | 0 | 0 | 0 | 1 | 0 |
| FLNB | 0 | 0 | 0 | 1 | 0 |
| VPS41 | 0 | 0 | 0 | 1 | 0 |
| PEAK1 | 0 | 0 | 0 | 1 | 0 |
| UBASH3B | 0 | 0 | 0 | 1 | 0 |
| TPBG | 0 | 0 | 0 | 1 | 0 |
| MSX2 | 0 | 0 | 0 | 1 | 0 |
| ADRB2 | 0 | 0 | 0 | 1 | 0 |
| C21orf58 | 0 | 0 | 0 | 1 | 0 |
| ILVBL | 0 | 0 | 0 | 1 | 0 |
| FOSL1 | 0 | 0 | 0 | 1 | 0 |
| TNFAIP3 | 0 | 0 | 0 | 1 | 0 |
| CCT7 | 0 | 0 | 0 | 1 | 0 |
| SERBP1 | 0 | 0 | 0 | 1 | 0 |
| ILF2 | 0 | 0 | 0 | 1 | 0 |
| HDAC1 | 0 | 0 | 0 | 1 | 0 |
| KAT6A | 0 | 0 | 0 | 1 | 0 |
| PIK3CA | 0 | 0 | 0 | 1 | 0 |
| ELK1 | 0 | 0 | 0 | 1 | 0 |
| LIME1 | 0 | 0 | 0 | 1 | 0 |
| SYK | 0 | 0 | 0 | 1 | 0 |
| ASS1 | 0 | 0 | 0 | 1 | 0 |
| HNRNPD | 0 | 0 | 0 | 1 | 0 |
| PALM2-AKAP2 | 0 | 0 | 0 | 1 | 0 |
| TRIM23 | 0 | 0 | 0 | 1 | 0 |
| C21orf91 | 0 | 0 | 0 | 1 | 0 |
| USP54 | 0 | 0 | 0 | 1 | 0 |
| BLNK | 0 | 0 | 0 | 1 | 0 |
| PSMC1 | 0 | 0 | 0 | 1 | 0 |
| STRADB | 0 | 0 | 0 | 1 | 0 |
| UBQLN1 | 0 | 0 | 0 | 1 | 0 |
| PUM1 | 0 | 0 | 0 | 1 | 0 |
| PPP4R2 | 0 | 0 | 0 | 1 | 0 |
| MAGEB18 | 0 | 0 | 0 | 1 | 0 |
| GAD1 | 0 | 0 | 0 | 1 | 0 |
| PDCD6IP | 0 | 0 | 0 | 1 | 0 |
| NPM1 | 0 | 0 | 0 | 1 | 0 |
| PTP4A1 | 0 | 0 | 0 | 1 | 0 |
| TCTA | 0 | 0 | 0 | 1 | 0 |
| MSMO1 | 0 | 0 | 0 | 1 | 0 |
| NADK | 0 | 0 | 0 | 1 | 0 |
| KRT32 | 0 | 0 | 0 | 1 | 0 |
| PXN | 0 | 0 | 0 | 1 | 0 |
| PLK1S1 | 0 | 0 | 0 | 1 | 0 |
| CDC5L | 0 | 0 | 0 | 1 | 0 |
| SF3B4 | 0 | 0 | 0 | 1 | 0 |
| PRICKLE3 | 0 | 0 | 0 | 1 | 0 |
| HOXC8 | 0 | 0 | 0 | 1 | 0 |
| SYP | 0 | 0 | 0 | 1 | 0 |
| KIT | 0 | 0 | 0 | 1 | 0 |
| NFS1 | 0 | 0 | 0 | 1 | 0 |
| CUL4A | 0 | 0 | 0 | 1 | 0 |
| UBE2I | 0 | 0 | 0 | 1 | 1 |
| RAB8B | 0 | 0 | 0 | 1 | 0 |
| KLHL8 | 0 | 0 | 0 | 1 | 0 |
| SOS1 | 0 | 0 | 0 | 1 | 0 |
| SF3A3 | 0 | 0 | 0 | 1 | 0 |
| PPP3CA | 0 | 0 | 0 | 1 | 0 |
| UPF2 | 0 | 0 | 0 | 1 | 0 |
| USP39 | 0 | 0 | 0 | 1 | 0 |
| DIAPH1 | 0 | 0 | 0 | 1 | 0 |
| KIF22 | 0 | 0 | 0 | 1 | 0 |
| PRPF40A | 0 | 0 | 0 | 1 | 0 |
| FYN | 0 | 0 | 0 | 1 | 1 |
| AHNAK | 0 | 0 | 0 | 1 | 0 |
| ERBB2 | 0 | 0 | 0 | 1 | 0 |
| ATP6V1B1 | 0 | 0 | 0 | 1 | 0 |
| VCL | 0 | 0 | 0 | 1 | 0 |
| TCP1 | 0 | 0 | 0 | 1 | 0 |
| ALG2 | 0 | 0 | 0 | 1 | 0 |
| CAD | 0 | 0 | 0 | 1 | 0 |
| HSPG2 | 0 | 0 | 0 | 1 | 0 |
| CRY2 | 0 | 0 | 0 | 1 | 0 |
| KRT86 | 0 | 0 | 0 | 1 | 0 |
| BTD | 0 | 0 | 0 | 1 | 0 |
| IRS2 | 0 | 0 | 0 | 1 | 0 |
| IDH3G | 0 | 0 | 0 | 1 | 0 |
| WDR6 | 0 | 0 | 0 | 1 | 0 |
| PEPD | 0 | 0 | 0 | 1 | 0 |
| MUC1 | 0 | 0 | 0 | 1 | 0 |
| NDUFA2 | 0 | 0 | 0 | 1 | 0 |
| ANP32A | 0 | 0 | 0 | 1 | 0 |
| RAE1 | 0 | 0 | 0 | 1 | 0 |
| BPGM | 0 | 0 | 0 | 1 | 0 |
| TIMM50 | 0 | 0 | 0 | 1 | 0 |
| PPAT | 0 | 0 | 0 | 1 | 0 |
| SPRY4 | 0 | 0 | 0 | 1 | 0 |
| BAZ2A | 0 | 0 | 0 | 1 | 0 |
| GABARAP | 0 | 0 | 0 | 1 | 0 |
| APOL5 | 0 | 0 | 0 | 1 | 0 |
| MED28 | 0 | 0 | 0 | 1 | 0 |
| ZXDC | 0 | 0 | 0 | 1 | 0 |
| CASC3 | 0 | 0 | 0 | 1 | 0 |
| ELMO2 | 0 | 0 | 0 | 1 | 0 |
| TRAF6 | 0 | 0 | 0 | 1 | 0 |
| MYO18A | 0 | 0 | 0 | 1 | 0 |
| CTTN | 0 | 0 | 0 | 1 | 0 |
| EIF5 | 0 | 0 | 0 | 1 | 0 |
| MYLK | 0 | 0 | 0 | 1 | 0 |
| NUTM2B | 0 | 0 | 0 | 1 | 0 |
| HMGN2 | 0 | 0 | 0 | 1 | 0 |
| ZCCHC13 | 0 | 0 | 0 | 1 | 0 |
| VASP | 0 | 0 | 0 | 1 | 0 |
| PBXIP1 | 0 | 0 | 0 | 1 | 0 |
| AKAP6 | 0 | 0 | 0 | 1 | 0 |
| ASXL1 | 0 | 0 | 0 | 1 | 0 |
| KRT9 | 0 | 0 | 0 | 1 | 0 |
| RBPMS | 0 | 0 | 0 | 1 | 0 |
| PTPRT | 0 | 0 | 0 | 1 | 0 |
| SNAPC3 | 0 | 0 | 0 | 1 | 0 |
| DLGAP1 | 0 | 0 | 0 | 1 | 0 |
| FLNC | 0 | 0 | 0 | 1 | 0 |
| FGD6 | 0 | 0 | 0 | 1 | 0 |
| YAP1 | 0 | 0 | 0 | 1 | 0 |
| KRT4 | 0 | 0 | 0 | 1 | 0 |
| GRIP2 | 0 | 0 | 0 | 1 | 0 |
| GBA | 0 | 0 | 0 | 1 | 0 |
| EPS15L1 | 0 | 0 | 0 | 1 | 0 |
| EXTL3 | 0 | 0 | 0 | 1 | 0 |
| USP30 | 0 | 0 | 0 | 1 | 0 |
| ZBTB16 | 0 | 0 | 0 | 1 | 0 |
| ABL2 | 0 | 0 | 0 | 1 | 0 |
| MAGEB2 | 0 | 0 | 0 | 1 | 0 |
| SH3PXD2B | 0 | 0 | 0 | 1 | 0 |
| KCNB2 | 0 | 0 | 0 | 1 | 0 |
| GNS | 0 | 0 | 0 | 1 | 0 |
| SIRPA | 0 | 0 | 0 | 1 | 0 |
| HNRPLL | 0 | 0 | 0 | 1 | 0 |
| TRIM32 | 0 | 0 | 0 | 1 | 0 |
| SREBF2 | 0 | 0 | 0 | 1 | 0 |
| RPS20 | 0 | 0 | 0 | 1 | 0 |
| SF3A2 | 0 | 0 | 0 | 1 | 0 |
| UBL4A | 0 | 0 | 0 | 1 | 0 |
| PAK1 | 0 | 0 | 0 | 1 | 0 |
| ECHS1 | 0 | 0 | 0 | 1 | 0 |
| CCT4 | 0 | 0 | 0 | 1 | 0 |
| HOOK3 | 0 | 0 | 0 | 1 | 0 |
| PLCG2 | 0 | 0 | 0 | 1 | 0 |
| CCL5 | 0 | 0 | 0 | 1 | 0 |
| LAX1 | 0 | 0 | 0 | 1 | 0 |
| MAP3K1 | 0 | 0 | 0 | 1 | 0 |
| SLC1A2 | 0 | 0 | 0 | 1 | 0 |
| AP4S1 | 0 | 0 | 0 | 1 | 0 |
| AKAP2 | 0 | 0 | 0 | 1 | 0 |
| GAREM | 0 | 0 | 0 | 1 | 0 |
| MET | 0 | 0 | 0 | 1 | 0 |
| KRT8 | 0 | 0 | 0 | 1 | 0 |
| RPP38 | 0 | 0 | 0 | 1 | 0 |
| FUS | 0 | 0 | 0 | 1 | 0 |
| SNX17 | 0 | 0 | 0 | 1 | 0 |
| SH3PXD2A | 0 | 0 | 0 | 1 | 0 |
| DRD4 | 0 | 0 | 0 | 1 | 0 |
| TCEANC | 0 | 0 | 0 | 1 | 0 |
| JAK1 | 0 | 0 | 0 | 1 | 0 |
| BTG3 | 0 | 0 | 0 | 1 | 0 |
| IK | 0 | 0 | 0 | 1 | 0 |
| BRCC3 | 0 | 0 | 0 | 1 | 0 |
| PSME1 | 0 | 0 | 0 | 1 | 0 |
| ETV4 | 0 | 0 | 0 | 1 | 0 |
| CHRNA7 | 0 | 0 | 0 | 1 | 0 |
| RNF10 | 0 | 0 | 0 | 1 | 0 |
| ACTN2 | 0 | 0 | 0 | 1 | 1 |
| DCTN2 | 0 | 0 | 0 | 1 | 1 |
| CCDC85B | 0 | 0 | 0 | 1 | 0 |
| CCT3 | 0 | 0 | 0 | 1 | 0 |
| SPRY2 | 0 | 0 | 0 | 1 | 0 |
| ENO1 | 0 | 0 | 0 | 1 | 0 |
| CHMP4A | 0 | 0 | 0 | 1 | 0 |
| ATP6V0D1 | 0 | 0 | 0 | 1 | 0 |
| RAI2 | 0 | 0 | 0 | 1 | 0 |
| GPR45 | 0 | 0 | 0 | 1 | 0 |
| SIGLEC7 | 0 | 0 | 0 | 1 | 0 |
| TCERG1 | 0 | 0 | 0 | 1 | 0 |
| SIX5 | 0 | 0 | 0 | 1 | 0 |
| GLTSCR1 | 0 | 0 | 0 | 1 | 0 |
| STK32C | 0 | 0 | 0 | 1 | 0 |
| PRPF3 | 0 | 0 | 0 | 1 | 0 |
| BASP1 | 0 | 0 | 0 | 1 | 0 |
| ARHGEF7 | 0 | 0 | 0 | 1 | 0 |
| PIK3R3 | 0 | 0 | 0 | 1 | 0 |
| CCKBR | 0 | 0 | 0 | 1 | 0 |
| CNOT1 | 0 | 0 | 0 | 1 | 0 |
| PIK3CG | 0 | 0 | 0 | 1 | 0 |
| APLP1 | 0 | 0 | 0 | 1 | 0 |
| QKI | 0 | 0 | 0 | 1 | 0 |
| ALAS2 | 0 | 0 | 0 | 1 | 0 |
| GCDH | 0 | 0 | 0 | 1 | 0 |
| DARS | 0 | 0 | 0 | 1 | 0 |
| EMID1 | 0 | 0 | 0 | 1 | 0 |
| CEP72 | 0 | 0 | 0 | 1 | 0 |
| WIPF3 | 0 | 0 | 0 | 1 | 0 |
| LOXL1 | 0 | 0 | 0 | 1 | 0 |
| PLD2 | 0 | 0 | 0 | 1 | 0 |
| HIPK3 | 0 | 0 | 0 | 1 | 0 |
| CSF1R | 0 | 0 | 0 | 1 | 0 |
| EIF2B4 | 0 | 0 | 0 | 1 | 0 |
| ZFYVE19 | 0 | 0 | 0 | 1 | 0 |
| DNAJB11 | 0 | 0 | 0 | 1 | 0 |
| ZC3H18 | 0 | 0 | 0 | 1 | 0 |
| OPCML | 0 | 0 | 0 | 1 | 0 |
| NCKIPSD | 0 | 0 | 0 | 1 | 0 |
| HIVEP1 | 0 | 0 | 0 | 1 | 0 |
| TTC9C | 0 | 0 | 0 | 1 | 0 |
| AMBP | 0 | 0 | 0 | 1 | 0 |
| STK39 | 0 | 0 | 0 | 1 | 0 |
| MARCH5 | 0 | 0 | 0 | 1 | 0 |
| EIF4G2 | 0 | 0 | 0 | 1 | 0 |
| MED15 | 0 | 0 | 0 | 1 | 0 |
| CAMK2B | 0 | 0 | 0 | 1 | 0 |
| ERRFI1 | 0 | 0 | 0 | 1 | 0 |
| RARS | 0 | 0 | 0 | 1 | 0 |
| BRCA2 | 0 | 0 | 0 | 1 | 0 |
| ABI3BP | 0 | 0 | 0 | 1 | 0 |
| NFASC | 0 | 0 | 0 | 1 | 0 |
| EHMT1 | 0 | 0 | 0 | 1 | 0 |
| RBM27 | 0 | 0 | 0 | 1 | 0 |
| PLXNB1 | 0 | 0 | 0 | 1 | 0 |
| ANKHD1-EIF4EBP3 | 0 | 0 | 0 | 1 | 0 |
| APBB1 | 0 | 0 | 0 | 1 | 0 |
| FRS2 | 0 | 0 | 0 | 1 | 0 |
| RIMS2 | 0 | 0 | 0 | 1 | 0 |
| RHOU | 0 | 0 | 0 | 1 | 0 |
| TRIM55 | 0 | 0 | 0 | 1 | 0 |
| ATF5 | 0 | 0 | 0 | 1 | 0 |
| TFAP2A | 0 | 0 | 0 | 1 | 0 |
| HOOK1 | 0 | 0 | 0 | 1 | 0 |
| PALM2 | 0 | 0 | 0 | 1 | 0 |
| SLX1A | 0 | 0 | 0 | 1 | 0 |
| HSFX1 | 0 | 0 | 0 | 1 | 0 |
| FLT3 | 0 | 0 | 0 | 1 | 0 |
| WDFY3 | 0 | 0 | 0 | 1 | 0 |
| PCM1 | 0 | 0 | 0 | 1 | 0 |
| POLR1D | 0 | 0 | 0 | 1 | 0 |
| HMGN3 | 0 | 0 | 0 | 1 | 0 |
| COIL | 0 | 0 | 0 | 1 | 0 |
| ADAM15 | 0 | 0 | 0 | 1 | 0 |
| PSPH | 0 | 0 | 0 | 1 | 0 |
| SHC2 | 0 | 0 | 0 | 1 | 0 |
| SMAD3 | 0 | 0 | 0 | 1 | 0 |
| HIST1H3F | 0 | 0 | 0 | 1 | 0 |
| SF1 | 0 | 0 | 0 | 1 | 0 |
| PAG1 | 0 | 0 | 0 | 1 | 0 |
| ADCY6 | 0 | 0 | 0 | 1 | 0 |
| HSPA4 | 0 | 0 | 0 | 1 | 0 |
| SF3B3 | 0 | 0 | 0 | 1 | 0 |
| SNRPB2 | 0 | 0 | 0 | 1 | 0 |
| GSTK1 | 0 | 0 | 0 | 1 | 0 |
| PHPT1 | 0 | 0 | 0 | 1 | 0 |
| RAPGEF4 | 0 | 0 | 0 | 1 | 0 |
| WBP4 | 0 | 0 | 0 | 1 | 0 |
| EIF4A3 | 0 | 0 | 0 | 1 | 0 |
| AJUBA | 0 | 0 | 0 | 1 | 0 |
| FOXJ1 | 0 | 0 | 0 | 1 | 0 |
| EIF1B | 0 | 0 | 0 | 1 | 0 |
| ZNF417 | 0 | 0 | 0 | 1 | 0 |
| ARHGAP32 | 0 | 0 | 0 | 1 | 0 |
| KRT10 | 0 | 0 | 0 | 1 | 0 |
| C2orf27A | 0 | 0 | 0 | 1 | 0 |
| HIST1H3C | 0 | 0 | 0 | 1 | 0 |
| EEF1A1P5 | 0 | 0 | 0 | 1 | 0 |
| CBLC | 0 | 0 | 0 | 1 | 0 |
| CCNK | 0 | 0 | 0 | 1 | 0 |
| SLC51A | 0 | 0 | 0 | 1 | 0 |
| ATP8 | 0 | 0 | 0 | 1 | 0 |
| PSTPIP2 | 0 | 0 | 0 | 1 | 0 |
| HIST1H4H | 0 | 0 | 0 | 1 | 0 |
| C2orf27B | 0 | 0 | 0 | 1 | 0 |
| GAB3 | 0 | 0 | 0 | 1 | 0 |
| EEF1D | 0 | 0 | 0 | 1 | 0 |
| HSPA1L | 0 | 0 | 0 | 1 | 0 |
| GAREML | 0 | 0 | 0 | 1 | 0 |
| NR4A1 | 0 | 0 | 0 | 1 | 0 |
| RAPGEF1 | 0 | 0 | 0 | 1 | 0 |
| MAP4K1 | 0 | 0 | 0 | 1 | 0 |
| FOXH1 | 0 | 0 | 0 | 1 | 0 |
| DLGAP4 | 0 | 0 | 0 | 1 | 0 |
| FAM160A2 | 0 | 0 | 0 | 1 | 0 |
| SDAD1 | 0 | 0 | 0 | 1 | 0 |
| CHRND | 0 | 0 | 0 | 1 | 0 |
| DOCK3 | 0 | 0 | 0 | 1 | 0 |
| ZNF276 | 0 | 0 | 0 | 1 | 0 |
| FLNA | 0 | 0 | 0 | 1 | 0 |
| PRKAR1A | 0 | 0 | 0 | 1 | 0 |
| LAPTM5 | 0 | 0 | 0 | 1 | 0 |
| PYCARD | 0 | 0 | 0 | 1 | 0 |
| SMURF1 | 0 | 0 | 0 | 1 | 0 |
| ASAP1 | 0 | 0 | 0 | 1 | 0 |
| CDKN1B | 0 | 0 | 0 | 1 | 0 |
| EPPK1 | 0 | 0 | 0 | 1 | 0 |
| ARHGAP17 | 0 | 0 | 0 | 1 | 0 |
| CD22 | 0 | 0 | 0 | 1 | 0 |
| PRRC2A | 0 | 0 | 0 | 1 | 0 |
| CBL | 0 | 0 | 0 | 1 | 0 |
| ATG5 | 0 | 0 | 0 | 1 | 0 |
| PHACTR2 | 0 | 0 | 0 | 1 | 0 |
| CSNK2B | 0 | 0 | 0 | 1 | 0 |
| HSPD1 | 0 | 0 | 0 | 1 | 0 |
| CLASRP | 0 | 0 | 0 | 1 | 0 |
| PLEKHB1 | 0 | 0 | 0 | 1 | 0 |
| PAK2 | 0 | 0 | 0 | 1 | 0 |
| KRT36 | 0 | 0 | 0 | 1 | 0 |
| HSPA8 | 0 | 0 | 0 | 1 | 0 |
| SEPN1 | 0 | 0 | 0 | 1 | 0 |
| MICAL1 | 0 | 0 | 0 | 1 | 0 |
| BCAR1 | 0 | 0 | 0 | 1 | 0 |
| TCEA1 | 0 | 0 | 0 | 1 | 0 |
| PLCG1 | 0 | 0 | 0 | 1 | 0 |
| ETV6 | 0 | 0 | 0 | 1 | 0 |
| FAM175B | 0 | 0 | 0 | 1 | 0 |
| F2RL2 | 0 | 0 | 0 | 1 | 0 |
| TNFRSF1A | 0 | 0 | 0 | 1 | 0 |
| KCNQ1 | 0 | 0 | 0 | 1 | 0 |
| CRIP2 | 0 | 0 | 0 | 1 | 0 |
| CXorf27 | 0 | 0 | 0 | 1 | 0 |
| DROSHA | 0 | 0 | 0 | 1 | 0 |
| SH3KBP1 | 0 | 0 | 0 | 1 | 0 |
| IQGAP1 | 0 | 0 | 0 | 1 | 0 |
| PSMC2 | 0 | 0 | 0 | 1 | 0 |
| DDX42 | 0 | 0 | 0 | 1 | 0 |
| CALM3 | 0 | 0 | 0 | 1 | 0 |
| PLEKHM1 | 0 | 0 | 0 | 1 | 0 |
| PNMA2 | 0 | 0 | 0 | 1 | 0 |
| TIMM44 | 0 | 0 | 0 | 1 | 0 |
| TOMM20 | 0 | 0 | 0 | 1 | 0 |
| CHRD | 0 | 0 | 0 | 1 | 0 |
| KRT83 | 0 | 0 | 0 | 1 | 0 |
| SUGP2 | 0 | 0 | 0 | 1 | 0 |
| RADIL | 0 | 0 | 0 | 1 | 0 |
| PTPRA | 0 | 0 | 0 | 1 | 0 |
| TEC | 0 | 0 | 0 | 1 | 0 |
| KCNN1 | 0 | 0 | 0 | 1 | 0 |
| YWHAH | 0 | 0 | 0 | 1 | 0 |
| MEPE | 0 | 0 | 0 | 1 | 0 |
| CWC25 | 0 | 0 | 0 | 1 | 0 |
| CCR10 | 0 | 0 | 0 | 1 | 0 |
| NAP1L5 | 0 | 0 | 0 | 1 | 0 |
| HIST1H4E | 0 | 0 | 0 | 1 | 0 |
| VAV2 | 0 | 0 | 0 | 1 | 0 |
| UBQLN4 | 0 | 0 | 0 | 1 | 0 |
| DRG1 | 0 | 0 | 0 | 1 | 0 |
| ATXN1L | 0 | 0 | 0 | 1 | 0 |
| PLRG1 | 0 | 0 | 0 | 1 | 0 |
| NARS | 0 | 0 | 0 | 1 | 0 |
| SH3BP2 | 0 | 0 | 0 | 1 | 0 |
| ZNF274 | 0 | 0 | 0 | 1 | 0 |
| CPSF7 | 0 | 0 | 0 | 1 | 0 |
| XAB2 | 0 | 0 | 0 | 1 | 0 |
| SGMS1 | 0 | 0 | 0 | 1 | 0 |
| KDR | 0 | 0 | 0 | 1 | 0 |
| FAM21C | 0 | 0 | 0 | 1 | 0 |
| AHCYL1 | 0 | 0 | 0 | 1 | 0 |
| YWHAB | 0 | 0 | 0 | 1 | 0 |
| PTPRJ | 0 | 0 | 0 | 1 | 0 |
| KRT82 | 0 | 0 | 0 | 1 | 0 |
| PPIB | 0 | 0 | 0 | 1 | 0 |
| NCOR1 | 0 | 0 | 0 | 1 | 0 |
| AIP | 0 | 0 | 0 | 1 | 0 |
| FAM193B | 0 | 0 | 0 | 1 | 0 |
| ZAP70 | 0 | 0 | 0 | 1 | 0 |
| PSMD7 | 0 | 0 | 0 | 1 | 0 |
| IL34 | 0 | 0 | 0 | 1 | 0 |
| FDPS | 0 | 0 | 0 | 1 | 0 |
| ELK3 | 0 | 0 | 0 | 1 | 0 |
| RASA1 | 0 | 0 | 0 | 1 | 0 |
| RCN1 | 0 | 0 | 0 | 1 | 0 |
| LASP1 | 0 | 0 | 0 | 1 | 0 |
| TULP1 | 0 | 0 | 0 | 1 | 0 |
| ABL1 | 0 | 0 | 0 | 1 | 0 |
| DOCK4 | 0 | 0 | 0 | 1 | 0 |
| VDAC1 | 0 | 0 | 0 | 1 | 0 |
| DMPK | 0 | 0 | 0 | 1 | 0 |
| TUBG1 | 0 | 0 | 0 | 1 | 0 |
| SPEN | 0 | 0 | 0 | 1 | 0 |
| CD59 | 0 | 0 | 0 | 1 | 0 |
| ARHGAP12 | 0 | 0 | 0 | 1 | 0 |
| FAM46A | 0 | 0 | 0 | 1 | 0 |
| EIF2B1 | 0 | 0 | 0 | 1 | 0 |
| NGFR | 0 | 0 | 0 | 1 | 0 |
| GCA | 0 | 0 | 0 | 1 | 0 |
| MAP2K5 | 0 | 0 | 0 | 1 | 0 |
| SCP2 | 0 | 0 | 0 | 1 | 0 |
| DHX37 | 0 | 0 | 0 | 1 | 0 |
| LAMC3 | 0 | 0 | 0 | 1 | 0 |
| CIRBP | 0 | 0 | 0 | 1 | 0 |
| PDE6G | 0 | 0 | 0 | 1 | 0 |
| SLC24A1 | 0 | 0 | 0 | 1 | 0 |
| COPS6 | 0 | 0 | 0 | 1 | 0 |
| HSD17B4 | 0 | 0 | 0 | 1 | 0 |
| FAT4 | 0 | 0 | 0 | 1 | 0 |
| RPS19 | 0 | 0 | 0 | 1 | 0 |
| WBP11 | 0 | 0 | 0 | 1 | 0 |
| PTPRC | 0 | 0 | 0 | 1 | 0 |
| TUBA1C | 0 | 0 | 0 | 1 | 0 |
| UTP14A | 0 | 0 | 0 | 1 | 0 |
| CLU | 0 | 0 | 0 | 1 | 0 |
| SHANK3 | 0 | 0 | 0 | 1 | 0 |
| SLC6A13 | 0 | 0 | 0 | 1 | 0 |
| SF3B1 | 0 | 0 | 0 | 1 | 0 |
| RHOXF2 | 0 | 0 | 0 | 1 | 0 |
| SH3D19 | 0 | 0 | 0 | 1 | 0 |
| MAPK1 | 0 | 0 | 0 | 1 | 0 |
| PRNP | 0 | 0 | 0 | 1 | 0 |
| ATXN2 | 0 | 0 | 0 | 1 | 0 |
| SEPT9 | 0 | 0 | 0 | 1 | 0 |
| DAB2 | 0 | 0 | 0 | 1 | 0 |
| MCM6 | 0 | 0 | 0 | 1 | 0 |
| PPP5C | 0 | 0 | 0 | 1 | 0 |
| SGOL1 | 0 | 0 | 0 | 1 | 0 |
| DLX4 | 0 | 0 | 0 | 1 | 0 |
| SLC4A2 | 0 | 0 | 0 | 1 | 0 |
| ATG12 | 0 | 0 | 0 | 1 | 0 |
| ITGA2B | 0 | 0 | 0 | 1 | 0 |
| IGF2R | 0 | 0 | 0 | 1 | 0 |
| NKD2 | 0 | 0 | 0 | 1 | 0 |
| BATF2 | 0 | 0 | 0 | 1 | 0 |
| NUMBL | 0 | 0 | 0 | 1 | 0 |
| HDAC5 | 0 | 0 | 0 | 1 | 0 |
| MTNR1A | 0 | 0 | 0 | 1 | 0 |
| CUTA | 0 | 0 | 0 | 1 | 0 |
| FTH1 | 0 | 0 | 0 | 1 | 0 |
| TF | 0 | 0 | 0 | 1 | 0 |
| BCR | 0 | 0 | 0 | 1 | 0 |
| RIMS1 | 0 | 0 | 0 | 1 | 0 |
| PRG4 | 0 | 0 | 0 | 1 | 0 |
| TFAP2B | 0 | 0 | 0 | 1 | 0 |
| KRT35 | 0 | 0 | 0 | 1 | 0 |
| AFF2 | 0 | 0 | 0 | 1 | 0 |
| LRSAM1 | 0 | 0 | 0 | 1 | 0 |
| UBAP2L | 0 | 0 | 0 | 1 | 0 |
| CCDC90B | 0 | 0 | 0 | 1 | 0 |
| HIBCH | 0 | 0 | 0 | 1 | 0 |
| EPB41 | 0 | 0 | 0 | 1 | 0 |
| HIST4H4 | 0 | 0 | 0 | 1 | 0 |
| TEK | 0 | 0 | 0 | 1 | 0 |
| IGFL3 | 0 | 0 | 0 | 1 | 0 |
| POMP | 0 | 0 | 0 | 1 | 0 |
| ACAP1 | 0 | 0 | 0 | 1 | 0 |
| U2AF1 | 0 | 0 | 0 | 1 | 0 |
| SHKBP1 | 0 | 0 | 0 | 1 | 0 |
| TRIP6 | 0 | 0 | 0 | 1 | 0 |
| PIN4 | 0 | 0 | 0 | 1 | 0 |
| TNIK | 0 | 0 | 0 | 1 | 0 |
| HIST1H3I | 0 | 0 | 0 | 1 | 0 |
| CLYBL | 0 | 0 | 0 | 1 | 0 |
| MCC | 0 | 0 | 0 | 1 | 0 |
| VSNL1 | 0 | 0 | 0 | 1 | 0 |
| NR1H4 | 0 | 0 | 0 | 1 | 0 |
| SART1 | 0 | 0 | 0 | 1 | 0 |
| RBM33 | 0 | 0 | 0 | 1 | 0 |
| THRAP3 | 0 | 0 | 0 | 1 | 0 |
| KRT6A | 0 | 0 | 0 | 1 | 0 |
| PRSS1 | 0 | 0 | 0 | 1 | 0 |
| U2AF2 | 0 | 0 | 0 | 1 | 0 |
| KRT33B | 0 | 0 | 0 | 1 | 0 |
| ITIH4 | 0 | 0 | 0 | 1 | 0 |
| DRD3 | 0 | 0 | 0 | 1 | 0 |
| HIST1H3B | 0 | 0 | 0 | 1 | 0 |
| HNRNPM | 0 | 0 | 0 | 1 | 0 |
| REPS1 | 0 | 0 | 0 | 1 | 0 |
| SH2B2 | 0 | 0 | 0 | 1 | 0 |
| MSI2 | 0 | 0 | 0 | 1 | 0 |
| DDX5 | 0 | 0 | 0 | 1 | 0 |
| TRAT1 | 0 | 0 | 0 | 1 | 0 |
| IL6ST | 0 | 0 | 0 | 1 | 0 |
| SNX8 | 0 | 0 | 0 | 1 | 0 |
| RPUSD2 | 0 | 0 | 0 | 1 | 0 |
| NCOR2 | 0 | 0 | 0 | 1 | 0 |
| APP | 0 | 0 | 0 | 1 | 0 |
| SNX7 | 0 | 0 | 0 | 1 | 0 |
| WDR44 | 0 | 0 | 0 | 1 | 0 |
| SYNCRIP | 0 | 0 | 0 | 1 | 0 |
| TTYH2 | 0 | 0 | 0 | 1 | 0 |
| VPS4A | 0 | 0 | 0 | 1 | 0 |
| RAB6B | 0 | 0 | 0 | 1 | 0 |
| IGFL2 | 0 | 0 | 0 | 1 | 0 |
| UHRF2 | 0 | 0 | 0 | 1 | 0 |
| SARNP | 0 | 0 | 0 | 1 | 0 |
| YBEY | 0 | 0 | 0 | 1 | 0 |
| METTL17 | 0 | 0 | 0 | 1 | 0 |
| LOC505709 | 0 | 0 | 0 | 1 | 0 |
| FHL2 | 0 | 0 | 0 | 1 | 0 |
| RAB1C | 0 | 0 | 0 | 1 | 0 |
| BAALC | 0 | 0 | 0 | 1 | 0 |
| INPPL1 | 0 | 0 | 0 | 1 | 0 |
| UBE2D2 | 0 | 0 | 0 | 1 | 0 |
| IGHM | 0 | 0 | 0 | 1 | 0 |
| RRAS | 0 | 0 | 0 | 1 | 0 |
| MAGOH | 0 | 0 | 0 | 1 | 0 |
| HIST1H3D | 0 | 0 | 0 | 1 | 0 |
| SLC25A5 | 0 | 0 | 0 | 1 | 0 |
| DOK3 | 0 | 0 | 0 | 1 | 0 |
| HDGFRP3 | 0 | 0 | 0 | 1 | 0 |
| CLPX | 0 | 0 | 0 | 1 | 0 |
| VSTM2L | 0 | 0 | 0 | 1 | 0 |
| EPOR | 0 | 0 | 0 | 1 | 0 |
| TUBA4B | 0 | 0 | 0 | 1 | 0 |
| USP34 | 0 | 0 | 0 | 1 | 0 |
| ADD3 | 0 | 0 | 0 | 1 | 0 |
| ID4 | 0 | 0 | 0 | 1 | 0 |
| SMEK1 | 0 | 0 | 0 | 1 | 0 |
| TIMM23 | 0 | 0 | 0 | 1 | 0 |
| CERS4 | 0 | 0 | 0 | 1 | 0 |
| DLGAP2 | 0 | 0 | 0 | 1 | 0 |
| ARHGEF5 | 0 | 0 | 0 | 1 | 0 |
| IRF9 | 0 | 0 | 0 | 1 | 0 |
| SELL | 0 | 0 | 0 | 1 | 0 |
| LY6G6F | 0 | 0 | 0 | 1 | 0 |
| PSMD11 | 0 | 0 | 0 | 1 | 0 |
| IRS4 | 0 | 0 | 0 | 1 | 0 |
| FCGR2C | 0 | 0 | 0 | 1 | 0 |
| AGT | 0 | 0 | 0 | 1 | 0 |
| RALGPS1 | 0 | 0 | 0 | 1 | 0 |
| MFAP4 | 0 | 0 | 0 | 1 | 0 |
| TGOLN2 | 0 | 0 | 0 | 1 | 0 |
| SEMA7A | 0 | 0 | 0 | 1 | 0 |
| CIC | 0 | 0 | 0 | 1 | 0 |
| SNRPA1 | 0 | 0 | 0 | 1 | 0 |
| HIST1H4J | 0 | 0 | 0 | 1 | 0 |
| GAPVD1 | 0 | 0 | 0 | 1 | 0 |
| MYH9 | 0 | 0 | 0 | 1 | 0 |
| MYRIP | 0 | 0 | 0 | 1 | 0 |
| H2AFX | 0 | 0 | 0 | 1 | 0 |
| FGFR1 | 0 | 0 | 0 | 1 | 0 |
| CNTNAP1 | 0 | 0 | 0 | 1 | 0 |
| KRT14 | 0 | 0 | 0 | 1 | 0 |
| AIM2 | 0 | 0 | 0 | 1 | 0 |
| SOCS1 | 0 | 0 | 0 | 1 | 0 |
| HELZ | 0 | 0 | 0 | 1 | 0 |
| RIT2 | 0 | 0 | 0 | 1 | 0 |
| SNRPD2 | 0 | 0 | 0 | 1 | 0 |
| KHDRBS1 | 0 | 0 | 0 | 1 | 0 |
| CASP2 | 0 | 0 | 0 | 1 | 0 |
| CCNA1 | 0 | 0 | 0 | 1 | 0 |
| MLL4 | 0 | 0 | 0 | 1 | 0 |
| ABI1 | 0 | 0 | 0 | 1 | 0 |
| SUV39H2 | 0 | 0 | 0 | 1 | 0 |
| ANKHD1 | 0 | 0 | 0 | 1 | 0 |
| GATAD1 | 0 | 0 | 0 | 1 | 0 |
| BCAR3 | 0 | 0 | 0 | 1 | 0 |
| MLLT4 | 0 | 0 | 0 | 1 | 0 |
| PSMB1 | 0 | 0 | 0 | 1 | 0 |
| GPANK1 | 0 | 0 | 0 | 1 | 0 |
| P2RX7 | 0 | 0 | 0 | 1 | 0 |
| USP8 | 0 | 0 | 0 | 1 | 0 |
| SIT1 | 0 | 0 | 0 | 1 | 0 |
| ZNF609 | 0 | 0 | 0 | 1 | 0 |
| TCEAL8 | 0 | 0 | 0 | 1 | 0 |
| KRT73 | 0 | 0 | 0 | 1 | 0 |
| UNK | 0 | 0 | 0 | 1 | 0 |
| HIST1H4C | 0 | 0 | 0 | 1 | 0 |
| PPP2R2A | 0 | 0 | 0 | 1 | 0 |
| IRF5 | 0 | 0 | 0 | 1 | 0 |
| NUP214 | 0 | 0 | 0 | 1 | 0 |
| PRPF19 | 0 | 0 | 0 | 1 | 0 |
| ACAT1 | 0 | 0 | 0 | 1 | 0 |
| FANCA | 0 | 0 | 0 | 1 | 0 |
| SEMA4G | 0 | 0 | 0 | 1 | 0 |
| DHRSX | 0 | 0 | 0 | 1 | 0 |
| PSMA6 | 0 | 0 | 0 | 1 | 0 |
| NISCH | 0 | 0 | 0 | 1 | 0 |
| IL3RA | 0 | 0 | 0 | 1 | 0 |
| NEB | 0 | 0 | 0 | 1 | 0 |
| LCP2 | 0 | 0 | 0 | 1 | 0 |
| EIF1AY | 0 | 0 | 0 | 1 | 0 |
| ELP5 | 0 | 0 | 0 | 1 | 0 |
| HOOK2 | 0 | 0 | 0 | 1 | 0 |
| RNF31 | 0 | 0 | 0 | 1 | 0 |
| PTK2B | 0 | 0 | 0 | 1 | 0 |
| UBR4 | 0 | 0 | 0 | 1 | 0 |
| FGFR2 | 0 | 0 | 0 | 1 | 0 |
| COX6A1 | 0 | 0 | 0 | 1 | 0 |
| OCRL | 0 | 0 | 0 | 1 | 0 |
| IKBKE | 0 | 0 | 0 | 1 | 0 |
| TOLLIP | 0 | 0 | 0 | 1 | 0 |
| TMX2 | 0 | 0 | 0 | 1 | 0 |
| FLT1 | 0 | 0 | 0 | 1 | 0 |
| SRP54 | 0 | 0 | 0 | 1 | 0 |
| WDR77 | 0 | 0 | 0 | 1 | 0 |
| BRD4 | 0 | 0 | 0 | 1 | 0 |
| SH2D1A | 0 | 0 | 0 | 1 | 0 |
| EPHB2 | 0 | 0 | 0 | 1 | 0 |
| SHC4 | 0 | 0 | 0 | 1 | 0 |
| KIAA2026 | 0 | 0 | 0 | 1 | 0 |
| ZNF488 | 0 | 0 | 0 | 1 | 0 |
| TCAP | 0 | 0 | 0 | 1 | 0 |
| ZC3H7B | 0 | 0 | 0 | 1 | 0 |
| NCAM1 | 0 | 0 | 0 | 1 | 0 |
| SLC25A51 | 0 | 0 | 0 | 1 | 0 |
| ZBTB7C | 0 | 0 | 0 | 1 | 0 |
| UBE2N | 0 | 0 | 0 | 1 | 0 |
| HTRA2 | 0 | 0 | 0 | 1 | 0 |
| HSFX2 | 0 | 0 | 0 | 1 | 0 |
| EEF1G | 0 | 0 | 0 | 1 | 0 |
| HP | 0 | 0 | 0 | 1 | 0 |
| MAP4K3 | 0 | 0 | 0 | 1 | 0 |
| WNK2 | 0 | 0 | 0 | 1 | 0 |
| HCN2 | 0 | 0 | 0 | 1 | 0 |
| HIST2H4B | 0 | 0 | 0 | 1 | 0 |
| SUMF1 | 0 | 0 | 0 | 1 | 0 |
| ZHX3 | 0 | 0 | 0 | 1 | 0 |
| VAV1 | 0 | 0 | 0 | 1 | 0 |
| POM121 | 0 | 0 | 0 | 1 | 0 |
| OR10B1P | 0 | 0 | 0 | 1 | 0 |
| ACOT7 | 0 | 0 | 0 | 1 | 0 |
| TM9SF4 | 0 | 0 | 0 | 1 | 0 |
| UBE2D1 | 0 | 0 | 0 | 1 | 0 |
| DHX16 | 0 | 0 | 0 | 1 | 0 |
| SREBF1 | 0 | 0 | 0 | 1 | 0 |
| DLGAP3 | 0 | 0 | 0 | 1 | 0 |
| TIMM17A | 0 | 0 | 0 | 1 | 0 |
| PPP6R2 | 0 | 0 | 0 | 1 | 0 |
| TBC1D5 | 0 | 0 | 0 | 1 | 0 |
| NEU3 | 0 | 0 | 0 | 1 | 0 |
| EIF2B3 | 0 | 0 | 0 | 1 | 0 |
| HIST1H3E | 0 | 0 | 0 | 1 | 0 |
| KIRREL | 0 | 0 | 0 | 1 | 0 |
| DDX17 | 0 | 0 | 0 | 1 | 0 |
| UBE2D3 | 0 | 0 | 0 | 1 | 0 |
| DNAJA3 | 0 | 0 | 0 | 1 | 0 |
| PTPRN2 | 0 | 0 | 0 | 1 | 0 |
| ACTA1 | 0 | 0 | 0 | 1 | 0 |
| PRPF31 | 0 | 0 | 0 | 1 | 0 |
| DCTN1 | 0 | 0 | 0 | 1 | 0 |
| ATXN7 | 0 | 0 | 0 | 1 | 0 |
| ANXA2 | 0 | 0 | 0 | 1 | 0 |
| RIF1 | 0 | 0 | 0 | 1 | 0 |
| RBMS1 | 0 | 0 | 0 | 1 | 0 |
| MATN2 | 0 | 0 | 0 | 1 | 0 |
| TMPO | 0 | 0 | 0 | 1 | 0 |
| CBLB | 0 | 0 | 0 | 1 | 0 |
| HNRNPU | 0 | 0 | 0 | 1 | 0 |
| BIVM | 0 | 0 | 0 | 1 | 0 |
| MTAP | 0 | 0 | 0 | 1 | 0 |
| SKAP1 | 0 | 0 | 0 | 1 | 0 |
| CSN2 | 0 | 0 | 0 | 1 | 0 |
| CKS2 | 0 | 0 | 0 | 1 | 0 |
| PIK3R2 | 0 | 0 | 0 | 1 | 0 |
| DOPEY1 | 0 | 0 | 0 | 1 | 0 |
| CD164 | 0 | 0 | 0 | 1 | 0 |
| RBFOX2 | 0 | 0 | 0 | 1 | 0 |
| KIAA0408 | 0 | 0 | 0 | 1 | 0 |
| AHSG | 0 | 0 | 0 | 1 | 0 |
| GC | 0 | 0 | 0 | 1 | 0 |
| IMPDH2 | 0 | 0 | 0 | 1 | 0 |
| INPP5D | 0 | 0 | 0 | 1 | 0 |
| SMARCC1 | 0 | 0 | 0 | 1 | 0 |
| THY1 | 0 | 0 | 0 | 1 | 0 |
| RBM26 | 0 | 0 | 0 | 1 | 0 |
| DAZAP2 | 0 | 0 | 0 | 1 | 0 |
| SETD6 | 0 | 0 | 0 | 1 | 0 |
| PPP3CB | 0 | 0 | 0 | 1 | 0 |
| C6 | 0 | 0 | 0 | 1 | 0 |
| CTBP2 | 0 | 0 | 0 | 1 | 0 |
| TFG | 0 | 0 | 0 | 1 | 0 |
| YLPM1 | 0 | 0 | 0 | 1 | 0 |
| HIST1H4F | 0 | 0 | 0 | 1 | 0 |
| CSRP1 | 0 | 0 | 0 | 1 | 0 |
| EPHB6 | 0 | 0 | 0 | 1 | 0 |
| DAG1 | 0 | 0 | 0 | 1 | 0 |
| WASF1 | 0 | 0 | 0 | 1 | 0 |
| FAR1 | 0 | 0 | 0 | 1 | 0 |
| A2M | 0 | 0 | 0 | 1 | 0 |
| DCTN3 | 0 | 0 | 0 | 1 | 0 |
| QRICH1 | 0 | 0 | 0 | 1 | 0 |
| ZNF311 | 0 | 0 | 0 | 1 | 0 |
| ARAP1 | 0 | 0 | 0 | 1 | 0 |
| FAM46B | 0 | 0 | 0 | 1 | 0 |
| H1F0 | 0 | 0 | 0 | 1 | 0 |
| EIF6 | 0 | 0 | 0 | 1 | 0 |
| UBE2E3 | 0 | 0 | 0 | 1 | 0 |
| CNTFR | 0 | 0 | 0 | 1 | 0 |
| HDGF | 0 | 0 | 0 | 1 | 0 |
| MCM10 | 0 | 0 | 0 | 1 | 0 |
| MTERFD2 | 0 | 0 | 0 | 1 | 0 |
| HIST1H3A | 0 | 0 | 0 | 1 | 0 |
| NAA40 | 0 | 0 | 0 | 1 | 0 |
| ECT2 | 0 | 0 | 0 | 1 | 0 |
| C10orf2 | 0 | 0 | 0 | 1 | 0 |
| CCT2 | 0 | 0 | 0 | 1 | 0 |
| FHOD1 | 0 | 0 | 0 | 1 | 0 |
| NELFB | 0 | 0 | 0 | 1 | 0 |
| IGF2BP1 | 0 | 0 | 0 | 1 | 0 |
| ARHGAP35 | 0 | 0 | 0 | 1 | 0 |
| MTA3 | 0 | 0 | 0 | 1 | 0 |
| ZSCAN1 | 0 | 0 | 0 | 1 | 0 |
| CFL1 | 0 | 0 | 0 | 1 | 0 |
| RIN3 | 0 | 0 | 0 | 1 | 0 |
| PQBP1 | 0 | 0 | 0 | 1 | 0 |
| BTK | 0 | 0 | 0 | 1 | 0 |
| EXOC7 | 0 | 0 | 0 | 1 | 0 |
| DOCK1 | 0 | 0 | 0 | 1 | 0 |
| SLAIN2 | 0 | 0 | 0 | 1 | 0 |
| CSF3R | 0 | 0 | 0 | 1 | 0 |
| EIF3F | 0 | 0 | 0 | 1 | 0 |
| AHDC1 | 0 | 0 | 0 | 1 | 0 |
| LNX1 | 0 | 0 | 0 | 1 | 0 |
| RAD54L2 | 0 | 0 | 0 | 1 | 0 |
| PDGFRB | 0 | 0 | 0 | 1 | 0 |
| SRSF5 | 0 | 0 | 0 | 1 | 0 |
| KRT18 | 0 | 0 | 0 | 1 | 0 |
| SUPT16H | 0 | 0 | 0 | 1 | 0 |
| FUBP3 | 0 | 0 | 0 | 1 | 0 |
| WIPF2 | 0 | 0 | 0 | 1 | 0 |
| USP53 | 0 | 0 | 0 | 1 | 0 |
| ERBB4 | 0 | 0 | 0 | 1 | 0 |
| LNX2 | 0 | 0 | 0 | 1 | 0 |
| USP6NL | 0 | 0 | 0 | 1 | 0 |
| JAK2 | 0 | 0 | 0 | 1 | 0 |
| HSPA1A | 0 | 0 | 0 | 1 | 0 |
| SMARCC2 | 0 | 0 | 0 | 1 | 0 |
| IRS1 | 0 | 0 | 0 | 1 | 0 |
| PDIA2 | 0 | 0 | 0 | 1 | 0 |
| UBD | 0 | 0 | 0 | 1 | 0 |
| WIPF1 | 0 | 0 | 0 | 1 | 0 |
| CST3 | 0 | 0 | 0 | 1 | 0 |
| GJA9 | 0 | 0 | 0 | 1 | 0 |
| FGFR3 | 0 | 0 | 0 | 1 | 0 |
| NCKAP1 | 0 | 0 | 0 | 1 | 0 |
| RBBP6 | 0 | 0 | 0 | 1 | 0 |
| VTN | 0 | 0 | 0 | 1 | 0 |
| TRAF2 | 0 | 0 | 0 | 1 | 0 |
| SMARCD2 | 0 | 0 | 0 | 1 | 0 |
| SBDS | 0 | 0 | 0 | 1 | 0 |
| SYBU | 0 | 0 | 0 | 1 | 0 |
| HEY2 | 0 | 0 | 0 | 1 | 0 |
| SH2B3 | 0 | 0 | 0 | 1 | 0 |
| SPRY1 | 0 | 0 | 0 | 1 | 0 |
| CELF2 | 0 | 0 | 0 | 1 | 0 |
| AIRE | 0 | 0 | 0 | 1 | 0 |
| LRP8 | 0 | 0 | 0 | 1 | 0 |
| KRT2 | 0 | 0 | 0 | 1 | 0 |
| VPS26A | 0 | 0 | 0 | 1 | 0 |
| UBE4B | 0 | 0 | 0 | 1 | 0 |
| ACIN1 | 0 | 0 | 0 | 1 | 0 |
| SLC23A1 | 0 | 0 | 0 | 1 | 0 |
| PLEC | 0 | 0 | 0 | 1 | 0 |
| CD72 | 0 | 0 | 0 | 1 | 0 |
| SSSCA1 | 0 | 0 | 0 | 1 | 0 |
| F7 | 0 | 0 | 0 | 1 | 0 |
| PNRC1 | 0 | 0 | 0 | 1 | 0 |
| PRAP1 | 0 | 0 | 0 | 1 | 0 |
| MAGEB6 | 0 | 0 | 0 | 1 | 0 |
| CREM | 0 | 0 | 0 | 1 | 0 |
| EIF2S3 | 0 | 0 | 0 | 1 | 0 |
| NR5A1 | 0 | 0 | 0 | 1 | 0 |
| LITAF | 0 | 0 | 0 | 1 | 0 |
| SMU1 | 0 | 0 | 0 | 1 | 0 |
| RBFOX1 | 0 | 0 | 0 | 1 | 0 |
| PAX3 | 0 | 0 | 0 | 1 | 0 |
| SYNM | 0 | 0 | 0 | 1 | 0 |
| SYN1 | 0 | 0 | 0 | 1 | 0 |
| OTX2 | 0 | 0 | 0 | 1 | 0 |
| HIST1H4I | 0 | 0 | 0 | 1 | 0 |
| RPA1 | 0 | 0 | 0 | 1 | 0 |
| STUB1 | 0 | 0 | 0 | 1 | 0 |
| HNRNPR | 0 | 0 | 0 | 1 | 0 |
| PPIF | 0 | 0 | 0 | 1 | 0 |
| DUSP15 | 0 | 0 | 0 | 1 | 0 |
| PSMD13 | 0 | 0 | 0 | 1 | 0 |
| CKAP5 | 0 | 0 | 0 | 1 | 0 |
| MYEF2 | 0 | 0 | 0 | 1 | 0 |
| OSGEP | 0 | 0 | 0 | 1 | 0 |
| KCNAB1 | 0 | 0 | 0 | 0 | 1 |
| KCNA2 | 0 | 0 | 0 | 0 | 1 |
| KCNA5 | 0 | 0 | 0 | 0 | 1 |
| KCNA4 | 0 | 0 | 0 | 0 | 1 |
| SUMO3 | 0 | 0 | 0 | 0 | 1 |
| TFRC | 0 | 0 | 0 | 0 | 1 |
| KCNA3 | 0 | 0 | 0 | 0 | 1 |
| DLG1 | 0 | 0 | 0 | 0 | 1 |
| DYNC1I1 | 0 | 0 | 0 | 0 | 1 |
| FHL1 | 0 | 0 | 0 | 0 | 1 |
| RAB4A | 0 | 0 | 0 | 0 | 1 |
